# The genes crucial to carotenoid metabolism under elevated CO_2_ levels in carrot (*Daucus carota* L.)

**DOI:** 10.1038/s41598-021-91522-7

**Published:** 2021-06-08

**Authors:** Hongxia Song, Qiang Lu, Leiping Hou, Meilan Li

**Affiliations:** Collaborative Innovation Center for Improving Quality and Increasing Profits of Protected Vegetables in Shanxi, College of Horticulture, Shanxi Agricultural University, Taigu, 030801 Shanxi People’s Republic of China

**Keywords:** Biological techniques, Molecular biology, Plant sciences

## Abstract

The CO_2_ saturation point can reach as high as 1819 μmol· mol^−1^ in carrot (*Daucus carota* L.). In recent years, carrot has been cultivated in out-of-season greenhouses, but the molecular mechanism of CO_2_ enrichment has been ignored, and this is a missed opportunity to gain a comprehensive understanding of this important process. In this study, it was found that CO_2_ enrichment increased the aboveground and belowground biomasses and greatly increased the carotenoid contents. Twenty genes related to carotenoids were discovered in 482 differentially expressed genes (DEGs) through RNA sequencing (RNA-Seq.). These genes were involved in either carotenoid biosynthesis or the composition of the photosystem membrane proteins, most of which were upregulated. We suspected that these genes were directly related to quality improvement and increases in biomass under CO_2_ enrichment in carrot. As such, β-carotene hydroxylase activity in carotenoid metabolism and the expression levels of coded genes were determined and analysed, and the results were consistent with the observed change in carotenoid content. These results illustrate the molecular mechanism by which the increase in carotenoid content after CO_2_ enrichment leads to the improvement of quality and biological yield. Our findings have important theoretical and practical significance.

## Introduction

Carrot (*Daucus carota* L. *var*. *sativa* D C.) belongs to the Umbelliferae family, is widely cultivated worldwide and is listed as one of the top ten produced vegetables in the world. Its carotenoid content is higher than that of other common vegetables, and thus, it is thought to have beneficial implications for nutrition, beauty, and cancer prevention^1^. Carotenoids are present widely in plants. The carotenoids in leaves act as antenna pigments, participate in photosynthesis and are responsible for the rich colours found in plant organs. Carotenoids are also precursors of plant hormones, which play a key role in plant growth and development and in cell membrane stability^2^.

In a controlled environment, CO_2_ fertigation enhances the photosynthetic rate and yield in both C3 and C4 crops^3^. The effect of CO_2_ enrichment on the carotenoid content of plants has been found to vary depending on the species. For example, some plants show an increase (e.g., *Solanum lycopersicum*, *Gyanura bicolor* and *Catharanthus roseus*), a decrease (e.g., *Glycine max*, *Zea mays*, *Brassica napus*, *Lactuca sativa*, *Populus tremuloides* and *Pinus ponderosa*), or no change (e.g., *Arabidopsis thaliana* and *Beta vulgaris*) in their carotenoid content in response to CO_2_ enrichment^4^. At present, the planting area of out-of-season facilities for carrots is gradually increasing, but few studies have investigated the effects of CO_2_ enrichment on yield and quality. Much research to date on carotenoids has focused mainly on the root, and it has been found that extreme CO_2_ concentrations inhibit the growth of carrot taproots^5^, but research on leaves is relatively rare^6^. In view of this, it is of great practical and theoretical value to study the mechanism by which carotenoid content changes in carrot leaves and roots following the application of elevated CO_2_ concentrations similar to those found in typical commercial greenhouses.

The synthesis and decomposition pathways of carotenoids are complex, but they are relatively conserved in plants, and the whole process is completed in the plastids^7^. The process is roughly divided into four steps and is regulated by a variety of enzymes^8^. The genes encoding the carotenoid metabolism-related enzymes have been cloned and expressed for different crops, but their expression patterns vary between species^9–11^. In one study, the relationship between the expression of carotenoid accumulation-related genes and their contents in five different coloured *Manihot esculenta* Crantz tubers was analysed using quantitative real-time PCR (RT-qPCR) and high-performance liquid chromatography (HPLC). The results showed that the accumulation of carotenoids is regulated by multiple genes, and there is a correlation between carotenoid content and root color^12^. In another study, the expression of carotenoid metabolism-related genes in tobacco leaves during senescence and maturation was analysed using transcriptome sequencing analysis combined with RT-qPCR, in which the expression of genes encoding enzymes involved in carotenoid synthesis was found to be downregulated, and the expression of genes encoding enzymes involved in carotenoid degradation was found to be upregulated^13^. Studies on carotenoids in carrots with different root colours have found that the accumulation of α-carotene and the formation of lutein may be related to the expression level of the carotene hydroxylase gene^14^. These results indicate that there are many kinds of carotenoids, and each enzyme in carotenoid metabolism may play a variable role depending on the environment or stage of development.

In this experiment, changes in carotenoid content and biological yield in carrots were measured. Carotenoid-related genes were screened using RNA sequencing (RNA-Seq.) technology. Carotenoid metabolism pathways, key enzyme activities, and changes in the expression of genes encoding enzymes involved in the metabolism of carotenoids were analysed in leaves under CO_2_ enrichment, and then the carotenoid mechanism was analysed under CO_2_ enrichment to lay a theoretical foundation for the scientific application of CO_2_ gas fertilizer in carrot cultivation.

## Results

### Effect of CO_2_ enrichment on biomass yield

**Figure 1:**
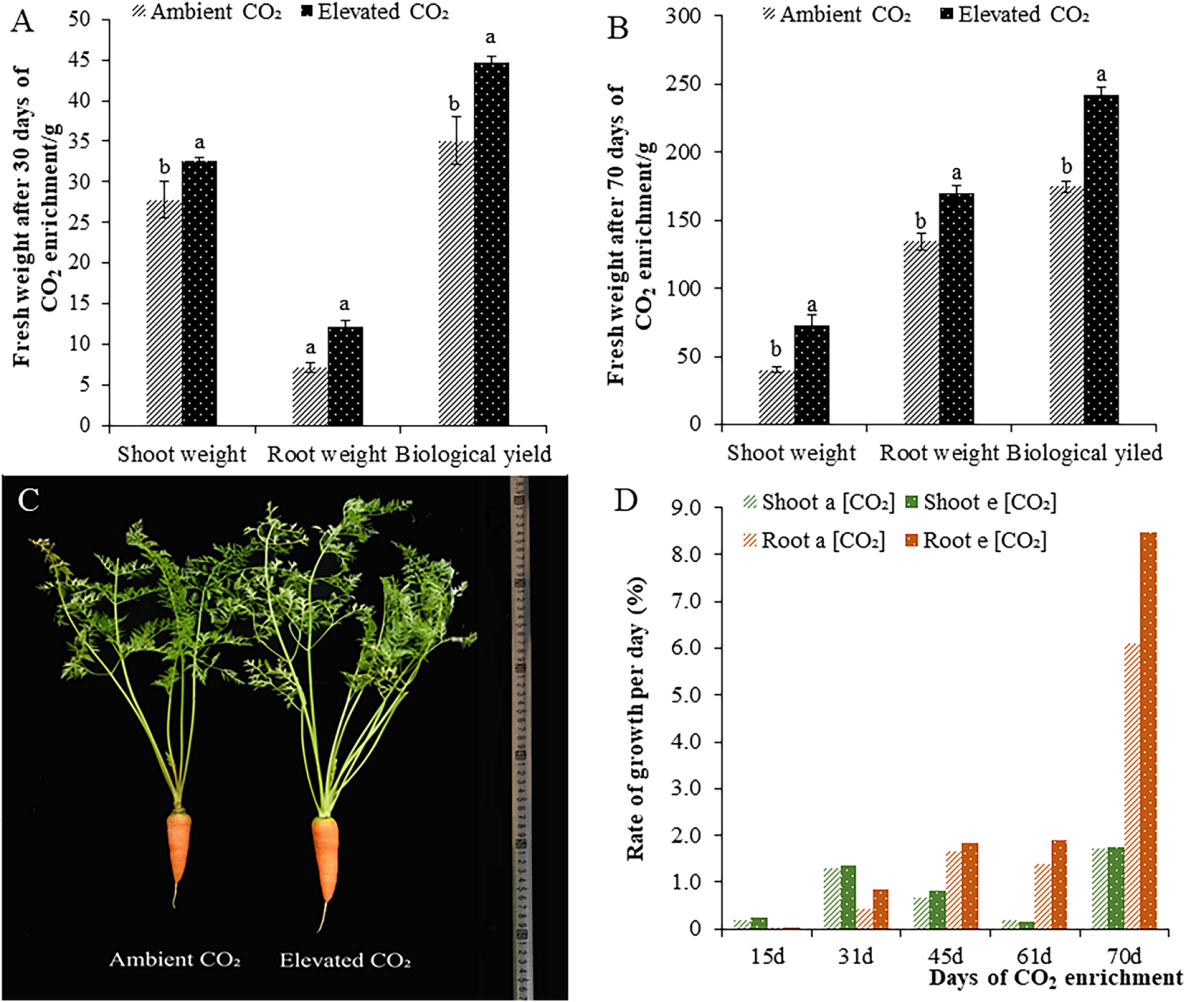
The effect of CO_2_ enrichment on the weight and growth rate of stems and roots. Biomass was measured 15, 31, 45, 61, and 70 days following the application of CO_2_. (C) 70 d after CO_2_ application. Capital letters in each figure and table represent extremely significant differences among samples by Student’s t-test (*P* < 0.01) and small letters represent significant differences (*P* < 0.05). Labels in the figures and tables below are the same.

The aboveground yields, belowground yields and total biomass yields of plants under CO_2_ enrichment were all significantly higher than those of the control at both 30 and 70 days following treatment (Fig. 1A, B, and C). The growth rates of shoots and roots were also compared, and CO_2_ enrichment significantly promoted the growth rate of roots at each stage. It is worth noting that between 15 and 31 days following CO_2_ treatment, the growth rates of aboveground organs were higher than those of belowground organs (Fig. 1D). This may be because the CO_2_ treatment first promoted the growth of the aboveground parts and then was transformed into the accumulation of underground nutrients.

### Effect of CO_2_ enrichment on carotenoid content

Under CO_2_ enrichment, the contents of four types of carotene in taproots increased, and the contents of α-carotene and β-carotene were significantly different from those in the control. All carotene contents increased in the leaves, the lutein content levels were similar to the levels of α-carotene content, but β-carotene contents were also higher. There was no significant difference in the ratio of chlorophyll to carotenoid content (Table 1).

**Table 1:** Effect of CO_2_ enrichment on carotenoids in carrot leaf and root.

		Lutein/μg·g^-1^ FW	Zeaxanthin/μg·g^-1^ FW	α- carotene /μg·g^-1^ FW	β-carotene/μg·g^-1^ FW	Chlorophyll/carotenoids
Root	Elevated CO_2_	5.38 ± 0.02 A	4.10 ± 0.04 A	198.84 ± 0.71 A	792.76 ± 6.92 A	–
	Ambient CO_2_	5.52 ± 0.10 A	3.82 ± 0.03 B	42.83 ± 0.25 B	232.89 ± 1.88 B	–
Leaf	Elevated CO_2_	101.11 ± 0.62 A	7.95 ± 0.08 A	96.53 ± 3.30 A	412.10 ± 2.20 A	1.77 ± 0.11 A
	Ambient CO_2_	69.36 ± 2.11 B	5.27 ± 0.19 B	58.42 ± 0.65 B	285.60 ± 1.46 B	2.54 ± 0.04 A

Sample were collected on 61 days after the initiation of the CO_2_ treatment.

### Sequencing quality assessment

The clean reads from each library were aligned to the carrot *Daucus carota* L. genome. Nearly 89.40%, 89.16%, and 89.69% of the control sample clean reads and 90.02%, 89.80%, and 89.57% of the CO_2_-enriched clean reads were annotated (Table 2). In these annotated reads, few cases of multiple reads corresponding to the same gene were observed, and most of the annotated genes had only one read (control sample: 82.72%, 82.23% and 83.02%; elevated CO_2_ sample: 82.52%, 82.80%, and 81.20%) with a high comparison efficiency. The results showed a high homology between carrot and the reference genome. Therefore, the selected reference genome was suitable for subsequent analysis.

**Table 2:** Sequence comparison of samples with reference genome.

	Clean reads	Mapped reads	Unique Mapped reads	Multiple Mapped reads
Elevated CO_2_-1	22,204,974	39,979,664 (90.02%)	36,644,873 (82.52%)	3,334,791 (7.51%)
Elevated CO_2_-2	24,809,680	44,443,819 (89.57%)	41,085,918 (82.80%)	3,357,901 (6.77%)
Elevated CO_2_-3	21,642,183	38,867,779 (89.80%)	35,148,471 (81.20%)	3,719,308 (8.59%)
Ambient CO_2_-1	26,650,192	47,649,902 (89.40%)	44,088,814(82.72%)	3,561,088 (6.68%)
Ambient CO_2_-2	26,920,393	48,004,141 (89.16%)	44,275,106 (82.23%)	3,729,035 (6.93%)
Ambient CO_2_-3	25,945,139	46,539,418 (89.69%)	43,077,476 (83.02%)	3,461,942 (6.67%)

### Repeated correlation assessment

**Figure 2:**
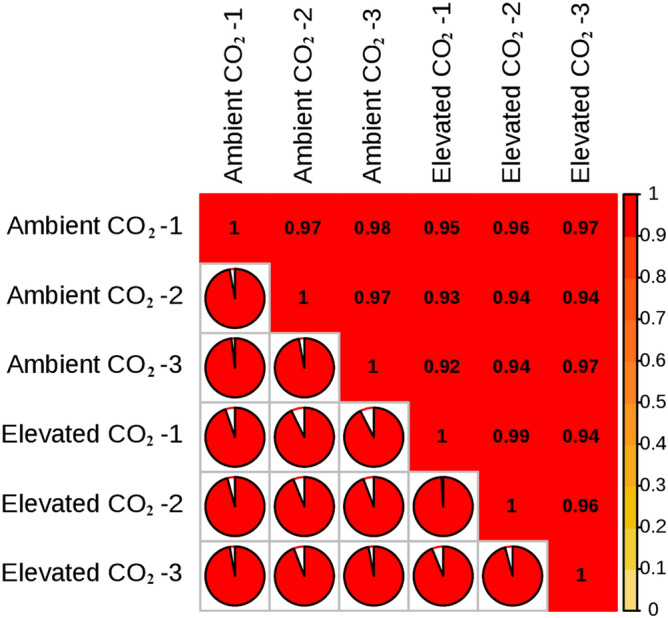
Repeated correlation analysis.

Transcriptome technology could not eliminate the variability due to the differences in gene expression in different individuals. To reduce the expression differences caused by individual biological variability and improve the reliability of differentially expressed genes, three biological replicates were used in the experimental design. According to the correlation analysis of transcriptome data (Fig. 2), the correlation R^2^ value among the three biological replicates of each treatment was above 0.88, which proves that the correlation between biological replicates was high.

### Screening of differentially expressed genes under CO_2_ enrichment

**Figure 3:**
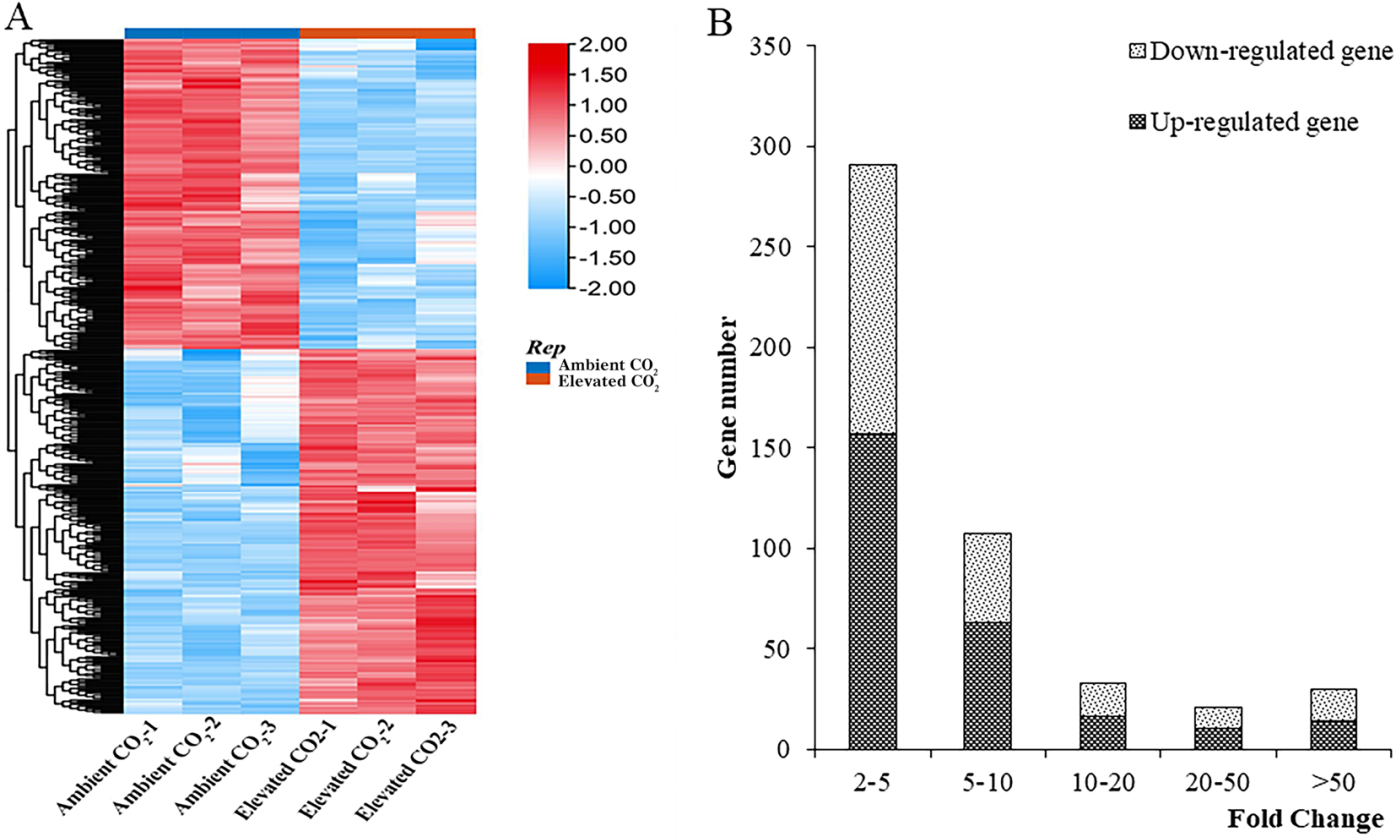
Statistical analysis of DEGs under CO_2_ enrichment in carrot. A false discovery rate (FDR) value ≤ 0.01 and a FC value ≥ 2 were used as thresholds to identify significant DEGs.

The number of differentially expressed genes (DEGs) between the control sample and the CO_2_-enriched sample was 482 (Supplementary Table S1), of which 260 were upregulated and 222 were downregulated (Fig. 3). The fold change (FC) was mainly two to five, and the number of upregulated and downregulated genes accounted for 60.38% and 60.36% of the total number of DEGs, respectively. A 5–10 FC of up- and downregulated genes in the DEGs accounted for 24.23% and 19.82% of the total number of DEGs, respectively. Up- and downregulated genes with a 10–20 FC totalled 16 and 17. The up- and downregulated DEGs with expression showing 20 FC or greater accounted for 3.85% and 4.96%, with 10 and 11 up- and downregulated genes, respectively. There were 14 and 16 up- and downregulated genes, respectively, whose expression levels differed by more than 50 FC.

### Screening of carotenoid-sensitive genes under CO_2_ enrichment

Using GO (Gene Ontology) annotation, 20 genes (Table 3) among the 482 DEGs were found to be directly related to carotenoids.

**Table 3:** DEGs related to carotenoid under elevated CO_2._

Gene ID	log_2_FC	Nr_annotation	Arabidopsis gene or annotation
*gene14276*	1.046155272	putative beta-ring carotene hydroxylase	*BCH-2*
*gene24757*	-1.64206568	linoleate 13S-lipoxygenase 2–1, chloroplastic like	*LOXs*
*gene946*	-3.183297791	linoleate 13S-lipoxygenase 2–1, chloroplastic-like	*LOXs*
*gene2572*	1.667625204	3-ketoacyl-CoA synthase 1	*KCS1*
*gene397*	-1.083773278	BnaA01g10370D [Brassica napus] U11/U12 small nuclear ribonucleoprotein 31 kDa protein	RNA-binding (RRM/RBD/RNP motifs) family protein
*gene33346*	2.63583798	photosystem I P700 apoprotein A2	*psaB*
*gene33347*	2.779902228	photosystem I P700 apoprotein A1	*psaA*
*gene33314*	2.13521141	photosystem II protein D1	*psbA*
*gene33382*	1.850928942	photosystem II CP47 chlorophyll apoprotein	*psbB*
*gene33340*	2.396875546	photosystem II CP43 chlorophyll apoprotein	*psbC*
*gene33339*	2.463811554	photosystem II protein D2	*psbD*
*gene33385*	2.294393093	photosystem II phosphoprotein	*psbH*
*gene23768*	2.680426839	photosystem II cp47 protein, partial (chloroplast)	*––-*
*gene33366*	1.442589023	cytochrome f	*petA*
*gene33386*	1.864735654	cytochrome b6	*petB*
*gene1293*	2.383516342	cytochrome b6 (chloroplast)	*––-*
*gene33327*	2.08889915	ATP synthase CF0 subunit IV	*atpI*
*gene15015*	-1.726685338	putative 9-cis epoxycarotenoid dioxygenase	*NCED-3*
*gene4178*	2.204143126	cytochrome P450 CYP707A67	*CYP707A1*
*gene1181*	-1.57780651	abscisic acid 8&apos-hydroxylase 4-like	*CYP707A4*

The expression of *gene14276* was upregulated, and its *Arabidopsis* homologous gene was *BCH-*2. BCH is one of the key enzymes in the upstream biosynthesis of zeaxanthin, which catalyses the synthesis of zeaxanthin from β-carotene by the intermediate product β-cryptoflavin. There are two *BCH* genes in *Arabidopsis*; their gene sequences are very similar, and the predicted proteins are nearly 70% homologous^15^. In this study, the expression of this gene was upregulated, indicating that CO_2_ enrichment promoted the formation of zeaxanthin. Davison et al.^16^ overexpressed the *AtBCH* gene in *Arabidopsis*, and the ability of transgenic *Arabidopsis* to resist abiotic stresses such as strong light, ultraviolet rays and high temperatures was significantly improved. After the expression of the *BCH* gene was inhibited, the carotenoid content decreased in *Arabidopsis*, and its tolerance to stress also decreased^15,17^.

All photosynthetic pigments and protein complexes involved in the photoreaction are located on the thylakoid membrane. The thylakoid membrane is composed of proteins, lipids, and pigments. Unsaturated fatty acid content, especially linolenic acid, is high in lipids. The expression of *gene24757* and *gene946* was downregulated, and the homologous *Arabidopsis* genes are the *LOXs*, which respond to high light intensity, jasmonic acid synthesis and lipid oxidation. The homologous *Arabidopsis* gene of *gene2572* is *KCS1*, which is involved in fatty acid biosynthesis. *Gene397* has carotenoid isomerase activity, and its homologous *Arabidopsis* gene is the RNA binding (RRM/RBD/RNP motif) family protein, which participates in mRNA cis-splicing and is located in chloroplasts.

The photosystem II (PSII) complex, photosystem I (PSI) complex, cytochrome b6f. (cytb 6F complex) and ATP synthase complex are the most important membrane protein complexes for photosynthesis^18^. *Gene33346* and *gene33347* are *psaB* and *psaA*, respectively; *psaB* and *psaA* are the basic polypeptide structures of the PSI photoreaction centre, and chlorophyll and β-carotene are combined with them. *Gene33314*, *gene33382*, *gene33340*, *gene33339*, *gene33385* and *gene23768* are the components of the PSII core complex; PSII binds many pigment molecules, including chlorophyll, β-carotene and lutein. The core antennas are composed of psbB and psbC, and psbA and psbD are the reaction centre proteins. PsbH is a subunit of cytochrome b559, and its function is unknown. *Gene33366*, *gene33386* and *gene1293* are all part of cytochrome b6f. (Cytb 6f. complex). Cytf, a component of the cytochrome b6f. complex, is involved in the electron transport process of photosynthesis in eukaryotes, connecting PSII and PSI electron flow, and plays an important role in photosynthesis^19^. *Gene33327* is the subunit of the CF0 unit of ATP synthase transmembrane. These 11 genes were upregulated after CO_2_ enrichment, which may promote photosynthesis in carrot.

The expression of *gene15015* was downregulated, and its homologous *Arabidopsis* gene is 9-cis-epoxy carotenoid dioxygenase (*NECD*). NECD is a rate-limiting enzyme that controls the transformation of carotenoids to ABA (abscisic acid), and it catalyses the cleavage of violaxanthin or neoxanthin to form the ABA precursor C15 xanthin^20^. It has been proven that the NCED protein is encoded by a multigene family, and the function and expression of each gene are different^21^. Its function needs to be further studied in carrot.

*Gene4178* and *gene1181* are both abscisic acid 8′-hydroxylases, and the number of 8′-hydroxylase family members varies from species to species: *Arabidopsis* has four (AtCYP707A1-4), and rice has three (OsABA8ox1-3)^22^. Transcripts of *Arabidopsis AtCYP707As* widely exist in various organs and tissues, but the expression levels are different. For example, the expression of *AtCYP-707A1* is highest in flowers and siliques, and *AtCYP707A2* and *AtCYP707A3* expression is highest in leaves, stems and roots, but *AtCYP707A4* is low in all tissues^23^. The expression patterns of the two genes in this study were different, which may also be related to the expression location and the expression level.

A comprehensive analysis of 20 genes showed that most of these genes were related to the biosynthesis of carotenoids or to the composition of the membrane protein of the photosystem, most of which were upregulated. This strongly indicates that CO_2_ enrichment promoted carotenoid metabolism, thereby enhancing carbon and nitrogen metabolism and promoting an increase in biomass.

### Expression analysis of genes encoding enzymes related to carotenoid metabolism

**Figure 4:**
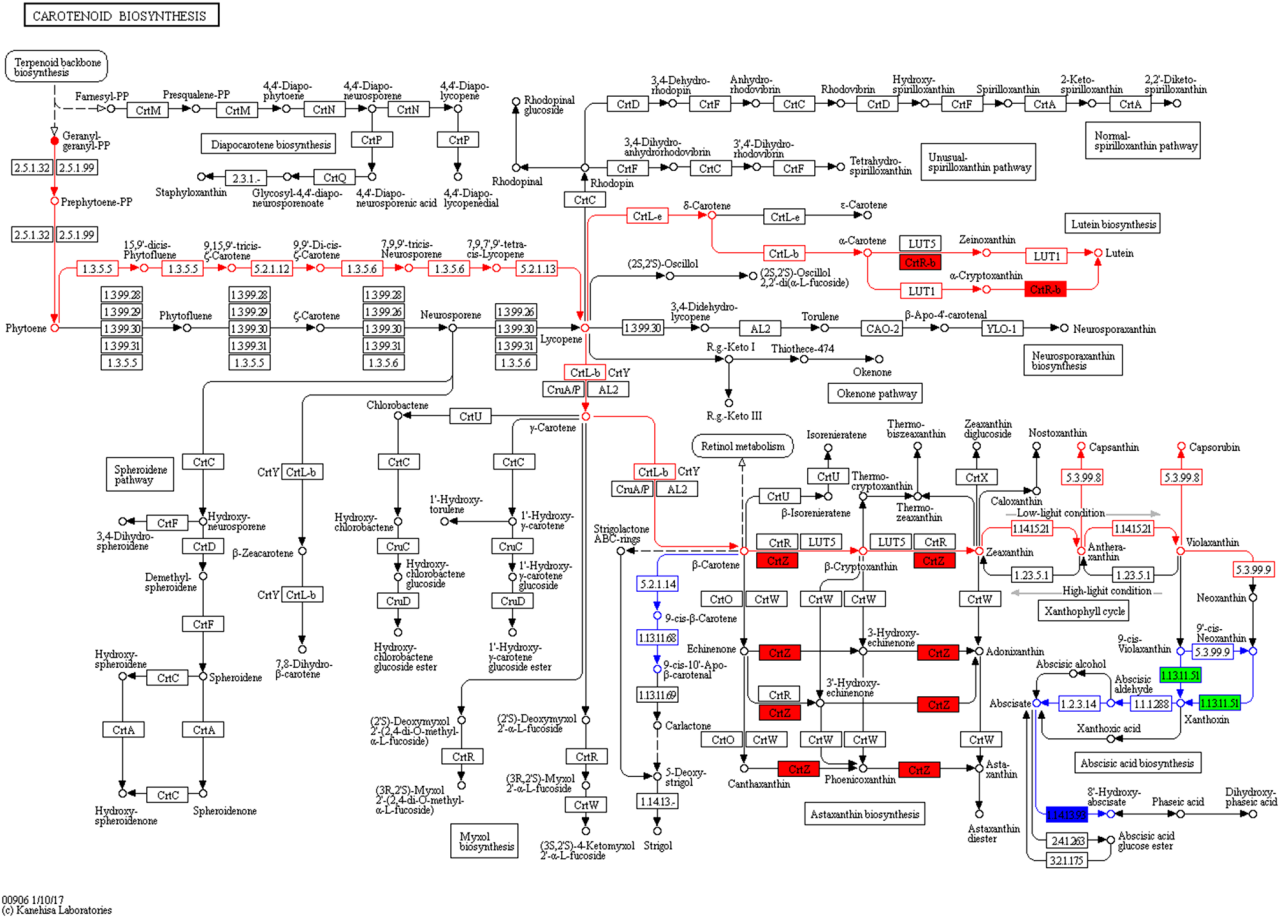
Carotenoid metabolism. Biosynthesis process is marked in red and the catabolism is labeled in blue. Using FC > 2 and FDR value ≤ 0.001 as the selection criteria to analyze the enrichment of enzymes.

A KEGG (https://www.kegg.jp/kegg/kegg1.html.) pathway map of carotenoid biosynthesis (KO00906) resulting from the RNA-seq. analysis is shown in Fig. 4.

Statistical analysis of the enzyme-encoded genes involved in carotenoid biosynthesis following CO_2_ enrichment showed that 12 enzymes were involved in carotenoid synthesis encoded by 20 genes (Table 4). Among them, only four were downregulated. These findings indicate that the carotenoid synthesis rate was significantly accelerated under CO_2_ enrichment, which was consistent with the observed increase in carotenoid content under CO_2_ enrichment.

**Table 4:** Enzymes and coded genes of carotenoid synthesis.

Enzyme ID	Enzyme	Coded gene in carrot	RPKM		Expression pattern
			Ambient CO_2_	Elevated CO_2_	
2.5.1.32	phytoene synthetase (PSY)	*gene20238/PSY-1*	126.27	140.02	Up
		*gene9917/PSY-2*	177.14	187.19	Up
		*gene24693/PSY-3*	0	0	–
1.3.5.5	phytoene desaturase (PDS)	*gene16125/PDS*	86.66	112.41	Up
		*DCAR_000109*	60.14	89.20	Up
5.2.1.12	ζ-carotene isomerase (Z-ISO)	*gene24145/Z-ISO*	59.19	89.20	Up
1.3.5.6	ζ-carotene desaturase (ZDS)	*DCAR_025321*	73.08	60.21	Down
		*gene6339/ZDS-1*	51.05	56.93	Up
5.2.1.13	prolycopene isomerase (CRTISO)	*gene15360/CRTISO-1*	41.16	41.40	Up
5.5.1.18	lycopene E- cyclase (CrtL-e)	*gene27498/LCYE*	68.27	77.12	Up
5.5.1.19	lycopene β- cyclase (CrtL-b)	*gene22843/LCYB1*	84.01	92.00	Up
1.14.99.45	carotenoid ε -hydroxylase (LUT1/CYP97C1)	*DCAR_017658*	23.94	28.17	Up
1.14.-.-	β-ring hydroxylase (LUT5/CYP97A3)	*DCAR_023843*	8.92	8.16	Down
1.14.13.129	β-carotene hydroxylase (crtZ/CrtR-b)	*gene14276/BCH-2*	21.47	42.40	Up
		*gene23124/BCH-1*	253.29	347.73	Up
		*DCAR_009395*	3.92	3.02	Down
1.14.15.21	zeaxanthin epoxidase (ZEP)	*DCAR_015695*	0	0.03	Up
		*gene26199/ZEP*	572.04	674.81	Up
1.23.5.1	violaxanthin de- epoxidase (VDE)	*DCAR_013654*	98.49	73.62	Down
5.3.99.8	capsanthin/capsorubin synthase (CCS1)	*DCAR_022896*	6.79	5.21	Down
5.3.99.9	neoxanthin synthase (NSY)	–	–	–	–

In carotenoid synthesis metabolism, there are six key rate-limiting enzymes: 2.5.1.32 (PSY), 5.5.1.18 (LCYe), 5.5.1.19 (LCYb), 1.14.99.45 (carotenoid epsilon hydroxylase, LUT1/CYP97C1), 1.14.-.- (beta-ring hydroxylase LUT5/CYP97A3) and 1.14.13.129 (BCH)^24^. Using a FC > 2 at an false discovery rate (FDR) ≤ 0.01 as the selection criteria, the KEGG pathway map was used to analyse the enrichment of enzymes in the carotenoid metabolism pathway, and we found that only 1.14.13.129 was enriched.

PSY is the core enzyme that determines the total carotenoid accumulation in plant tissues, according to the most in-depth study of carotenoid metabolism enzymes^25^. There are usually multiple *PSY* genes in plants, but only one has been found in *Arabidopsis*^26^ and three in tomato and cassava^27^. Not every *PSY* gene is related to carotenoid accumulation in fruits, and their expression is specific^28^. In this study, three *PSY* genes were found, and *PSY-1* and *PSY-2* were upregulated under CO_2_ enrichment.

The formation of α-carotene and β-carotene requires two lycopene cyclases (LCYb and LCYe). During citrus fruit ripening, the expression of the *LCYb* gene is upregulated, which promotes the conversion of lycopene to β-carotene and α-carotene^29,30^. The expression level of *LCYe* determines, to some extent, the ratio of carotenoids between β- and α-branches^17^. In this study, both LCYb and LCYe encoded a gene, both of which were upregulated. This indicates that CO_2_ enrichment promoted the transformation of lycopene to β-carotene and α-carotene. Moreover, the expression level of *LCYb1* was higher than that of *LCYe*, indicating that the synthesis of β-carotene may be slightly higher, which was consistent with the observed significant increase in β-carotene content under CO_2_ enrichment.

There are two types of hydroxylases, CHYB (BCH) and CYP97; of the latter type, hydroxylases CYP97A and CYP97C hydroxylate the β- and ε-rings, respectively. The orange-coloured α-carotene is sequentially catalysed primarily by CYP97-type hydroxylases to produce yellow lutein, and the orange-coloured β-carotene in the β, β-branch is hydroxylated by CHYB to produce yellow zeaxanthin. The enzyme CrtZ/CrtR-b belongs to the CHYB (BCH) type and encodes *BCH-2*, *BCH-1* and *DCAR_009395*, two of which are upregulated and one is downregulated. Among these, *BCH-2* was a significantly upregulated DEG, and its homologous *Arabidopsis* gene has been analysed in the section on the screening of carotenoid-sensitive genes under CO_2_ enrichment. LUT1 and LUT5 belong to the CYP97 type. Four hydroxylase genes were isolated from *Arabidopsis*. *CYP97A3*, the fourth hydroxylase gene, has higher biological activity on the β-ring of α-carotene but lower catalytic activity on the β-ring of β-carotene^31^. *CYP97A3* is more sensitive to strong light than *CYP97C1* and plays a synergistic role under different light intensities to promote lutein formation^32,33^. In this study, the expression of *DCAR_017658* encoding LUT1 (CYP97C1) was upregulated and that of *DCAR_023843* encoding LUT5 (CYP97A3) was downregulated, which indicates that the formation of lutein under CO_2_ enrichment might vary depending on environmental conditions. It also indicates that there was a competitive relationship between the two genes.

In the carotenoid degradation process, seven enzymes were found to be involved and encoded by 25 genes (Table 5). Among them, 1.13.11.51 (encoded by *gene15015*) and 1.14.13.93 (encoded by *gene1181* and *gene4178*) were significantly enriched by using FC ≥ 2 at an FDR value < 0.01 as the selection criteria (Fig. 4); *gene15015* and *gene1181* were downregulated and *gene4178* was upregulated, which indicates that catabolism occurred during carotenoid synthesis. Analysis of 25 genes encoding degrading enzymes showed that only six genes were upregulated, indicating that the decomposition efficiency of carotenoids was relatively slow under CO_2_ enrichment. Comparing the RPKM (Reads Per Kilobase of transcript per Million fragments mapped) values of all genes in carotenoid metabolism, the values of most genes in the degradation process were smaller than those in the synthesis process, which indicates that the synthesis of carotenoids was dominant in this study. The increase in carotenoid content under CO_2_ enrichment may be due to the gradual decrease in carotenoid degradation and the significant enhancement of the synthesis reaction. NECDs are the rate-limiting enzymes that control the conversion of carotenoids to ABA, and they are significantly upregulated during ageing^34^. In this study, *NCED-3* was significantly downregulated, which may be related to the leaf position we selected for sampling. CYP707 is a key enzyme for ABA decomposition and metabolism^23^ and is encoded by eight genes: *CYP707a-2* was significantly upregulated, *CYP707b-1* was significantly downregulated, and the expression of four genes was downregulated, indicating that *CYP707* directly inhibited ABA degradation, thereby reducing the degradation of carotenoids.

**Table 5:** Enzymes and coded genes of carotenoid degradation process.

Enzyme ID	Enzyme	Coded gene in carrot	RPKM		Expression pattern
			Ambient CO_2_	Elevated CO_2_	
1.13.11.51	nine- cis- epoxycarotenoid dioxygenase (NCED)	*gene15015/NCED-3*	4.13	1.17	Down
		*gene17667/NCED-2*	0.68	0.29	Down
		*gene1842/NCED-5*	0	0	–
		*gene19824/NCED-1*	1.06	0.98	Down
		*gene22296/NCED-9*	0	0	–
		*gene28051/NCED-8*	0.05	0.009	Down
		*gene31667/NCED4-CCD4*	304.69	240.16	Down
		*gene3531/NCED-6*	0.27	0.21	Down
		*gene4514/NCED-7*	0.14	0	Down
1.1.1.288	Xanthoxin dehydrogenase (ABA2)	*DCAR_005308*	7.89	6.40	Down
1.2.3.14	abscisic aldehyde oxidase (AAO3)	*DCAR_022906*	0.02	0.26	Up
		*DCAR_022905*	3.68	4.69	Up
		*DCAR_031841*	15.31	13.61	Down
1.14.13.93	abscisic acid 8'-hydroxylase (CYP707A)	*gene216/CYP707a-1*	0.35	0.60	Up
		*gene4178/CYP707a-2*	0.83	3.69	Up
		*gene1181/CYP707b-1*	7.80	2.52	Down
		*gene7668/CYP707b-2*	0.01	0	Down
		*gene10790/CYP707b-3*	0	0	–
		*gene22449/CYP707b-4*	5.92	4.49	Down
		*gene5663/CYP707c-1*	9.60	3.53	Down
		*gene7198/CYP707c-2*	0.55	0.01	Down
5.2.1.14	β-carotene isomerase	*DCAR_012509*	69.93	72.21	Up
		*DCAR_018439*	0.11	0.06	Down
1.13.11.68	9-cis-β-carotene 9', 10' cleavage dioxygenase (CCD7)	*gene32812/CCD7*	0.04	0.02	Down
1.13.11.69	carlactone synthase (CCD8)	*gene4112/CCD8*	0.01	0.06	Up

### The effect of CO_2_ enrichment on BCH activity

**Figure 5:**
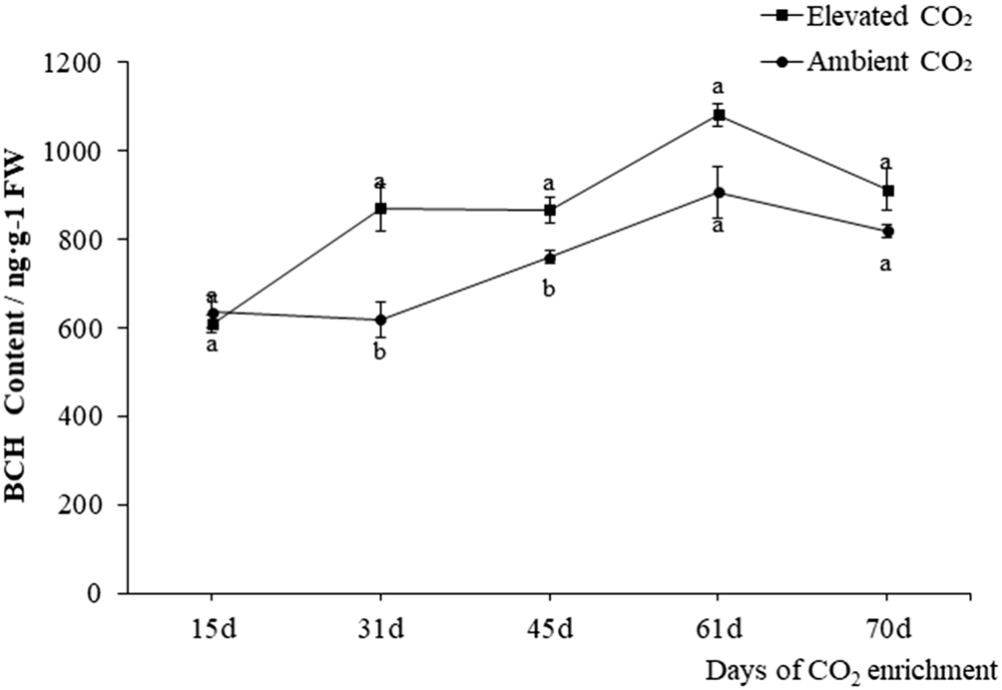
The effect of CO_2_ enrichment on carrot BCH enzyme activity. The sampling time was the same as the biomass measurement time, but samples were taken from other plants.

Analysis showed that 1.14.13.129 (BCH) was an enrichment enzyme in the KEGG pathway of carotenoid synthesis and was also a key rate-limiting enzyme in the carotenoid biosynthetic metabolic pathway. Under CO_2_ enrichment conditions, enzyme activity was always significantly higher than that of the control, and activity gradually increased as treatment progressed. The enzyme activity of plants in the control and the treatment peaked at 61 days and then slowly decreased (Fig. 5).

### Reverse transcription quantitative real-time PCR verification

**Figure 6:**
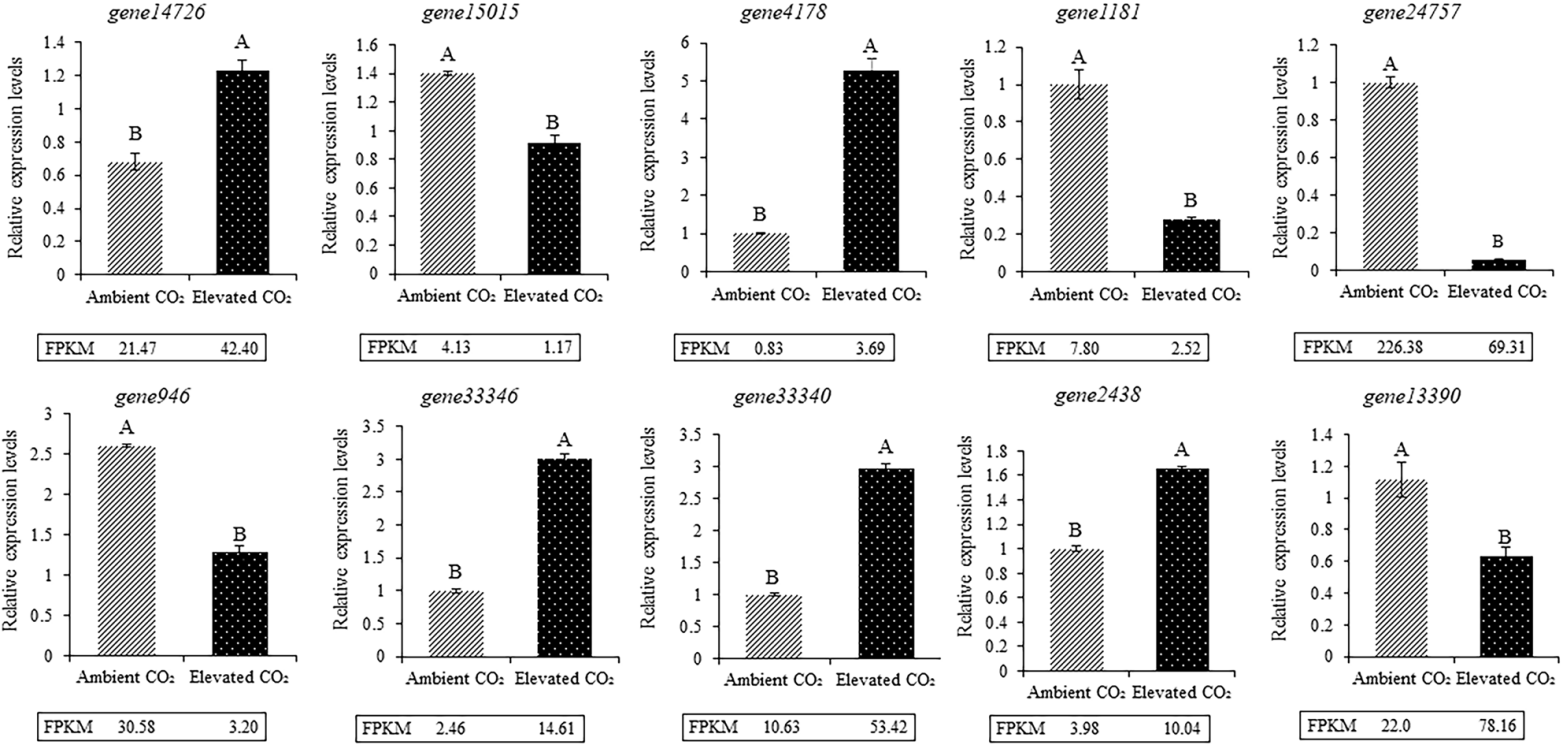
RT-qPCR validation of DGEs results based on gene expression levels.

Ten DEGs (*gene14276*, *gene15015*, *gene4178*, *gene1181*, *gene24757*, *gene946*, *gene33346*, *gene33340*, *gene2438*, and *gene13390*) were selected for RT-qPCR verification in plants under CO_2_ enrichment and control conditions to verify the reliability of the RNA-Seq. results (Fig. 6). Comparing the RT-qPCR results with the sequencing results, the expression trend in the 10 genes under CO_2_ enrichment was consistent with that of the sequencing results, indicating the reliability of the sequencing method. Among them, *gene14276* and *gene4178* were significantly upregulated, *gene15015* and *gene1181* were significantly downregulated in the carotenoid metabolic pathway (Fig. 6), and the expression trend of these four genes corresponded to the sequencing results.

## Discussion

Pigment content is the basis of carbon and nitrogen metabolism in plants and plays a positive role in promoting crop growth and development, yield, and quality^35^. In this study, the total biological yield increased following CO_2_ enrichment. In addition, at the initial stage following CO_2_ enrichment, aboveground biological yield increased faster than belowground yield. At the same time, the contents of carotenoids in leaves and roots increased, and the content of leaves was higher than that of roots. Our findings are consistent with Ma et al.^6^, although they found that the root contents were higher than the leaf contents following CO_2_ enrichment. It is unknown whether carotenoids in leaves transfer to fleshy roots following CO_2_ enrichment, and thus this needs further study. Zhang^36^ found that an appropriate increase in the CO_2_ concentration increases carotenoid content. This is conducive to the absorption of light energy by plants. We reached a similar conclusion; CO_2_ enrichment increased the carotenoid content in carrot, after which both the biological yield and root quality were significantly improved. In plants, an optimal level of carotenoid content is required to maintain environmental conditions for growth and development^4^. Lutein and β-carotene accumulate to form carotenoid lighting complexes. These carotenoids maintain the functional stability of the photosynthetic apparatus, and the ratio of chlorophyll to carotenoid must be strictly maintained^37^. Under CO_2_ enrichment, all carotene contents increased in leaves, lutein content level was similar to the level of α-carotene content, and β-carotene content was also higher. This indicates that CO_2_ enrichment promoted photosynthesis or enhanced light protection. There was no significant difference in the ratio of chlorophyll to carotenoid content, which is consistent with a study by Biswal^38^ where the pigment pool in leaves with photosynthetic activity was found to be stable before leaf senescence. In a study on the effects of CO_2_ application on *Arabidopsis thaliana* leaves at different leaf ages, it was found that increased CO_2_ levels causes the content of carotenoids in young leaves to increase^4^. This verifies the reliability of sampling the fourth leaf nearest the stem.

Most of the CO_2_ enrichment studies to date have used doubled CO_2_ concentrations, which promote crop production. When carrots are treated with higher concentrations of CO_2_, the taproot weight decreases, and CO_2_ enrichment inhibits taproot growth^5^. This may be due to higher CO_2_ concentrations inducing stoma closure and inhibiting CO_2_ fixation. The seedlings of two varieties of *Pinus koraiensis* were exposed to high concentrations of CO_2_ for one and a half years, after which they showed some stress symptoms, such as mottling, middle needle abscission, and early senescence^39^. All of these studies indicate that it is necessary to explore optimal concentrations and exposure periods of CO_2_ enrichment to benefit production.

The chlorophyll metabolism pathway is significantly affected under CO_2_ enrichment in cucumbers^40^. In this study, analysis of the KEGG pathway showed that the carotenoid metabolism pathway was significantly enriched (data not shown), and carotenoid content increased significantly under CO_2_ enrichment. Although chlorophyll content increased at the same time, the chlorophyll metabolism pathway did not reach the enrichment level. This may be due to the varied responses of different species and cultivation seasons to CO_2_ enrichment. In addition, the greenhouse in this study was covered with red spectroscopic film, which reduced the transmission ratio of visible light and increased the transmission ratio of ultraviolet, far-red light and near-infrared light, thus promoting photosynthesis in cucumber leaves.

Generally, the ratio of chlorophyll to carotenoids is 3:1. In this study, the ratio was smaller, which may have been caused by the increase in carotenoid content. The increase in carotenoids not only has a photoprotective function but can also be used for light harvesting^41^. This experiment was conducted in autumn and winter. During the experiment, there was no high temperature or strong light, so carotenoids mainly played the role of light capture in this study. Carrot is a crop that accumulates a high amount of carotenoids^5^. During the experiment, the red spectroscopic film covering the greenhouse may have also played a positive role in induction^42^.

To understand why there was an increase in biomass and carotenoid content in carrot under CO_2_ enrichment, 20 DEGs related to carotenoid metabolism were screened by transcriptome sequencing in this study. GO functional annotations were carried out on these DEGs. Five genes (*gene14276*, *gene24757*, *gene2572*, *gene397* and *gene946*) participated in the carotenoid biosynthetic process (GO: 0,016,117). The homologous *Arabidopsis* gene of *gene14276* is *BCH*, which encodes β-carotene hydroxylase. This enzyme is important in catalysing the synthesis of zeaxanthin from β-carotene via the intermediate product β-cryptoxanthin. Enzyme activity increased in all stages, and its coded gene expression was significantly upregulated under CO_2_ enrichment, which then promoted the synthesis of zeaxanthin. The results of ectopic expression of the moso bamboo *PeBCH* in *Arabidopsis* showed that the transgenic plants grow vigorously with increased chlorophyll, carotenoid and lutein contents^43^. The *crtRB1* gene in maize was overexpressed, which led to a 12.6-fold increase in β-carotene^44^. Compared with a control, after this gene was silenced in tobacco, the expression of the downstream violaxanthin deep oxidase gene and the zeaxanthin cyclooxygenase gene was significantly reduced, the β-carotene content was significantly increased, and the contents of violaxanthin and neoxanthin were significantly reduced^45^. Diretto et al.^46^ showed that inhibiting the expression of *BCH* affects the formation of carotenoids in potatoes, increasing the content of β-carotene to 38 times that of the original. In our study, enzyme activity began to decline 70 days after CO_2_ application; moreover, there was a clear decrease in the amplitude of samples from the CO_2_ treatment, and the specific reasons for this need further research.

Carotenoid metabolism is complicated, and there is a degradation reaction at the same time as synthesis. The net accumulation of carotenoids in plant tissues depends on the rate of biosynthesis and degradation. According to our pathway analysis, NCED (1.13.11.51) was significantly expressed in the degradation process. NCED is the rate-limiting enzyme controlling the transformation of carotenoids to ABA; the gene encoding this enzyme (*gene15015*) was verified by RT-qPCR analysis, and its expression was downregulated under CO_2_ enrichment. This indicates that under the experimental conditions, carotenoids were mainly synthesized, accompanied by their slow degradation. The fruit-specific RNAi-mediated *SlNCED1* inhibitor causes tomato fruits to produce a dark red colour, reduces *SlNCED1* transcription and ABA biosynthesis and increase the accumulation of lycopene and β-carotene^47^. *AcNCED1* silencing inhibits ABA synthesis and delays the softening of kiwifruit, while *AcNCED1* transient overexpression in tomato may accelerate the formation of fruit colour^48^. The NCED multigene family has a complex function, and the regulation of carotenoid metabolism needs further study.

## Materials and methods

### Experimental materials

The carrot inbred line ‘Tianhong No. 1–1’ was presented and licenced by the Carrot Breeding Team of College of Horticulture, Shanxi Agricultural University (Shanxi, China).

### Material processing

The experiment was conducted in a solar greenhouse at the Horticultural Station of Shanxi Agricultural University from September 2019 to January 2020, and the greenhouse in this study was covered with red spectroscopic film. The carbon-enriched zone (the CO_2_ concentration was 800 ± 50 µmol·mol^-1^, expressed hereafter as “elevated CO_2_”) and control zone (natural environment, expressed as “ambient CO_2_”) in the solar greenhouse were separated by a plastic film. The equipment and gas source used in the CO_2_ automatic release system were the same as those outlined in Song et al.^40^. On September 29, 2019, the seeds were sown in ridges; the width of each ridge was 40 cm, the ridge spacing was 50 cm, and the height of each ridge was 20 cm. CO_2_ treatment began on October 31, 2019, from 9:00 to 11:00 a.m. (on sunny days), and at this time, the seedling had four true leaves; treatment was paused on snowy days, and there were 48 days total for treatment. The plants were cultivated using traditional methods.

### Determination of biomass index

Taproot and shoot fresh weights and the total biomasses of the control and the treatment were measured 15, 31, 45, 61, and 70 days following the application of CO_2_. The experiment used 3 biological replicates per treatment, and 15 plants were sampled for each biological replication. In addition, the growth rates per day of stems and taproots were calculated as growth rates per day = (W_2_-W_1_)/(W_1_*D), where W_2_ was the quality of sampling in this measurement, W_1_ was the quality of sampling in last measurement, and D was the number of days between two samplings.

### Determination of carotenoid content

Three independent replications were used for each treatment, and there were 3 plants for each replication. On each plant, the fourth leaf and the phloem of the taproot were harvested. Approximately 0.2 g of sample was weighed, and carotenoids were extracted with acetone solution containing 0.1% BHT. The carotenoid content was determined using HPLC (2695 performance liquid chromatography, UPLC, Waters Company, USA). The UPLC column used was an Ultimate XB-30 (250 × 4.6 mm, 5 μm; Waters Corporation), the detection wavelength was 450 nm, the mobile phase was ethyl acetate: acetonitrile (1:1), and the flow rate was 1 mL· min^-1^. Standard samples of β-carotene, α-carotene, lutein, and zeaxanthin were purchased from Shanghai Yuanye Biotechnology Co., Ltd. (Shanghai, China).

### Sample collection

Samples were obtained at 10:00 a.m. on December 28, 2019 (a sunny day, 61 days after CO_2_ application). The fourth leaf of three healthy representative plants was selected and then combined into a biological replication; the test was repeated 3 times. Approximately 0.15 g of each sample was collected. After sampling, the specimen was immediately frozen in liquid nitrogen and stored at − 80 °C.

### Sample RNA extraction and detection

Total RNA was extracted from each sample using an RNeasy Plant Mini Kit (Qiagen, 74,903) following the manufacturer’s instructions. RNA integrity was determined by 1% agarose gel electrophoresis. The quality and quantity of the RNA were determined by the use of a NanoDrop 1000 spectrophotometer, and all samples showed 260/280 nm ratios of 2.0–2.1.

### cDNA library construction, sequence analysis and alignment

The mRNA from the total RNA samples was enriched using oligomagnetic adsorption, and the resulting RNA was fragmented. The RNA fragments served as a template for first-strand cDNA synthesis using random hexamers and reverse transcriptase. Second-strand cDNA was synthesized using DNA polymerase I and RNaseH and purified using a QiaQuick PCR extraction kit. Finally, cDNA fragments of a suitable length (300–500 bp) were obtained by agarose gel electrophoresis and amplified by PCR to construct the final cDNA libraries for paired-end sequencing using the Illumina HiSeq 2500 system (Biomarker Technologies Co., Ltd, Beijing, China)^49^. A total of 6 cDNA libraries were obtained. Raw reads from each sample were processed by removing rRNA and low-quality reads to obtain clean data (clean reads). The Q30 and GC contents of the clean data were also calculated. Downstream analyses were based on high-quality clean data. The clean reads from each library were aligned to the carrot *Daucus carota* L. genome (https://www.ncbi.nlm.nih.gov/assembly/GCF_001625215.1/) using HISAT2 (http://ccb.jhu.edu/software/hisat2/index.shtml). The aligned reads were assembled and quantified by StringTie (https://ccb.jhu.edu/software/stringtie/index.shtml.).

### Differential expression analysis of unigenes

The levels of gene expression in various samples were compared using the DESeq method, and an FDR value < 0.01 and an FC value ≥ 2 were used as thresholds to identify significant differentially expressed genes (DEGs). Hierarchical clustering of all DEGs was performed using R software (v 2.15.3) (https://cran.r-project.org/index.html) and displayed by Heatmap.

### Determination of key carotenoid enzyme activity

The sampling time was 15, 31, 45, 61, and 70 days after the application of CO_2_; three biological repetitions for each treatment were arranged, three plants were selected for each repetition, and only the fourth leaf was picked for each plant. BCH levels were determined using a plant enzyme-linked immunosorbent assay kit (Shanghai Jiang Lai Biological Technology Co., Ltd., Shanghai, China), and the operating method was completely in accordance with the manufacturer’s instructions.

### Reverse transcription quantitative real-time PCR

To validate the RNA sequencing results, RT-qPCR was performed using gene-specific primers for 10 selected genes (*gene14276*, *gene15015*, *gene4178*, *gene1181*, *gene24757*, *gene946*, *gene33346* and *gene33340*, which were involved in carotenoid metabolism, and *gene2438* and *gene13390*, which were randomly selected). Primer-BLAST (https://www.ncbi.nlm.nih.gov/tools/primer-blast/) was used to design specific primers, and details of the primer pairs are provided in Supplementary Table 2. The data were analysed by ABI 7500 software, and the reactions were carried out by the ABI 7500 Real-Time PCR System according to the manufacturer’s instructions as follows: 95 °C for 10 min, followed by 40 cycles at 94 °C for 15 s and 60 °C for 1 min, followed by melting curve analysis. The *ACTIN* gene has been identified as a suitable reference gene for the normalization of gene expression in carrot at different developmental stages^50^ and under abiotic stresses^51^. The *ACTIN* gene of carrot was chosen to normalize the expression levels of carotenoid biosynthesis and recycling genes in Tianhong No. 1–1 carrot cultivars under two CO_2_ concentration treatments. The sampling method and time were the same as those for the transcriptome, with 3 biological replicates for each test sample. The methods of reverse transcription and RT-qPCR were the same as those outlined in Sun et al.^49^, and the relative gene expression was calculated using the 2^−△△Ct^ method^52^. The values for the mean expression and standard deviation (SD) were calculated.

### Statistical analysis

Values represent the means ± one standard deviation SD of three replicates. The statistical analyses were analysed with one-way ANOVA and performed by the Statistical Analysis System (SAS, North Carolina, USA) with homoscedasticity instruction.

### Ethical statement

All local, national or international guidelines and legislation were adhered to in the production of this study.

## Supplementary Information

Supplementary Information 1.

Supplementary Information 2.
