# Supplementary material for: The genes crucial to carotenoid metabolism under elevated CO2 levels in carrot (Daucus carota L.)
Source: Sci Rep. 2021 Jun 8;11:12073. doi: 10.1038/s41598-021-91522-7 (PMC8187729; doi:10.1038/s41598-021-91522-7)
Supplement: Supplementary file 1 — Supplementary Information 1. [file 41598_2021_91522_MOESM1_ESM.pdf]

The Genes Crucial to Carotenoid Metabolism under Elevated CO_2 Levels in Carrot (*Daucus carota* L.)

Hongxia Song[†], Qiang Lu[†], Leiping Hou and Meilan Li*

Collaborative Innovation Center for Improving Quality and Increasing Profits of Protected Vegetables in Shanxi, College of Horticulture, Shanxi Agricultural University, Taigu, Shanxi, China

[†]These authors have contributed equally to this work

[*] Corresponding author:

Prof. Meilan Li.

E-mail: 15935485975@163.com

Institutional mail: College of Horticulture, Shanxi Agricultural University, Taigu District, Shanxi Province, P. R. China. Zip code: 030801

Supplementary table S1 482 DEG under elevated CO_2

ID	log_2FC	Nr_annotation
Daucus_carota_newGene_1020	-Inf	hypothetical protein Csa_6G504610 [Cucumis sativus]
Daucus_carota_newGene_1038	2.438884	PREDICTED: O-acyltransferase WSD1-like [Daucus carota subsp. sativus]
Daucus_carota_newGene_1066	-5.13289	PREDICTED: uncharacterized protein LOC108218155 [Daucus carota subsp. sativus]
Daucus_carota_newGene_1068	-4.65958	PREDICTED: uncharacterized protein LOC108218155 [Daucus carota subsp. sativus]
Daucus_carota_newGene_1069	2.614314	PREDICTED: uncharacterized protein LOC108217763 [Daucus carota subsp. sativus]
Daucus_carota_newGene_1072	2.831327	PREDICTED: protein argonaute 1-like [Daucus carota subsp. sativus]
Daucus_carota_newGene_1073	2.341181	PREDICTED: protein argonaute 1-like [Daucus carota subsp. sativus]
Daucus_carota_newGene_1076	3.019947	PREDICTED: protein argonaute 1-like [Daucus carota subsp. sativus]
Daucus_carota_newGene_1097	1.019709	PREDICTED: putative lipase YOR059C [Daucus carota subsp. sativus]
Daucus_carota_newGene_1125	2.172615	PREDICTED: actin-depolymerizing factor 12-like isoform X1 [Daucus carota subsp. sativus]
Daucus_carota_newGene_1136	-1.66931	PREDICTED: acyltransferase-like protein At3g26840, chloroplastic isoform X2 [Daucus carota subsp. sativus]
Daucus_carota_newGene_1658	4.625578	PREDICTED: uncharacterized protein LOC108222348 [Daucus carota subsp. sativus]
Daucus_carota_newGene_1791	-2.64522	PREDICTED: protein LNK2 isoform X2 [Daucus carota subsp. sativus]
Daucus_carota_newGene_1890	-1.30892	PREDICTED: probable disease resistance protein At4g27220 [Daucus carota subsp. sativus]
Daucus_carota_newGene_2058	2.677135	PREDICTED: lysine-specific demethylase JMJ25-like [Daucus carota subsp. sativus]
Daucus_carota_newGene_2163	-2.20187	PREDICTED: sesquiterpene synthase 2 [Daucus carota subsp. sativus]
Daucus_carota_newGene_2474	-Inf	PREDICTED: uncharacterized protein LOC108217084 [Daucus carota subsp. sativus]
Daucus_carota_newGene_2602	1.060662	PREDICTED: transcription factor GTE7-like [Daucus carota subsp. sativus]
Daucus_carota_newGene_2603	-4.78057	PREDICTED: deacetylvindoline O-acetyltransferase-like [Daucus carota subsp. sativus]
Daucus_carota_newGene_2607	-2.14347	PREDICTED: protein TPLATE-like [Daucus carota subsp. sativus]
Daucus_carota_newGene_2635	5.69099	PREDICTED: uncharacterized protein LOC109237086 [Nicotiana attenuata]
Daucus_carota_newGene_2733	-1.20876	--
Daucus_carota_newGene_2791	-Inf	PREDICTED: uncharacterized protein LOC108199480 [Daucus carota subsp. sativus]
Daucus_carota_newGene_2792	-1.85049	PREDICTED: probable E3 ubiquitin-protein ligase XERICO [Daucus carota subsp. sativus]
Daucus_carota_newGene_2810	2.554956	PREDICTED: uncharacterized protein LOC108205936 isoform X2 [Daucus carota subsp. sativus]
Daucus_carota_newGene_2837	1.056841	PREDICTED: thioredoxin-like protein AAED1, chloroplastic [Daucus carota subsp. sativus]
Daucus_carota_newGene_2847	1.615095	PREDICTED: pyruvate kinase 1, cytosolic isoform X1 [Daucus carota subsp. sativus]
Daucus_carota_newGene_2986	-1.50846	PREDICTED: putative glycine-rich cell wall structural protein 1 [Daucus carota subsp. sativus]
Daucus_carota_newGene_30	2.76298	PREDICTED: IMPACT family member in pol 5'region isoform X2 [Daucus carota subsp. sativus]
Daucus_carota_newGene_3057	1.431099	PREDICTED: uncharacterized protein LOC108194362 [Daucus carota subsp. sativus]

Daucus_carota_newGene_3058	3.154265	PREDICTED: uncharacterized protein LOC108194362 [Daucus carota subsp. sativus]
Daucus_carota_newGene_3122	-1.00077	--
Daucus_carota_newGene_3175	2.424414	PREDICTED: protein AUXIN-REGULATED GENE INVOLVED IN ORGAN SIZE-like [Daucus carota subsp. sativus]
Daucus_carota_newGene_318	2.888518	hypothetical protein DCAR_001031 [Daucus carota subsp. sativus]
Daucus_carota_newGene_3245	4.792673	--
Daucus_carota_newGene_3260	3.140392	--
Daucus_carota_newGene_330	-3.65266	PREDICTED: uncharacterized protein LOC108217438 [Daucus carota subsp. sativus]
Daucus_carota_newGene_3423	-2.4146	PREDICTED: protein NUCLEAR FUSION DEFECTIVE 4-like [Daucus carota subsp. sativus]
Daucus_carota_newGene_3440	3.95108	PREDICTED: uncharacterized protein LOC108195441 [Daucus carota subsp. sativus]
Daucus_carota_newGene_3469	-2.54401	hypothetical protein DCAR_025138 [Daucus carota subsp. sativus]
Daucus_carota_newGene_365	-1.94417	--
Daucus_carota_newGene_3795	2.55141	hypothetical protein DCAR_028912 [Daucus carota subsp. sativus]
Daucus_carota_newGene_4176	1.05518	PREDICTED: delta(12) fatty acid desaturase FAD2-like [Daucus carota subsp. sativus]
Daucus_carota_newGene_4233	-3.91833	--
Daucus_carota_newGene_4332	-Inf	--
Daucus_carota_newGene_4355	-3.39697	PREDICTED: kirola-like [Daucus carota subsp. sativus]
Daucus_carota_newGene_4371	-2.4136	PREDICTED: lipid phosphate phosphatase 1-like isoform X1 [Daucus carota subsp. sativus]
Daucus_carota_newGene_4382	-1.23376	PREDICTED: GDT1-like protein 4 [Daucus carota subsp. sativus]
Daucus_carota_newGene_4391	3.933408	PREDICTED: uncharacterized protein LOC108197552 [Daucus carota subsp. sativus]
Daucus_carota_newGene_4612	-2.76797	PREDICTED: GDSL esterase/lipase At1g54790 [Daucus carota subsp. sativus]
Daucus_carota_newGene_4618	2.760842	--
Daucus_carota_newGene_4732	1.575843	PREDICTED: cysteine-rich receptor-like protein kinase 42 [Daucus carota subsp. sativus]
Daucus_carota_newGene_4736	1.457868	--
Daucus_carota_newGene_4882	3.094731	PREDICTED: uncharacterized protein LOC108201836 isoform X1 [Daucus carota subsp. sativus]
Daucus_carota_newGene_5179	4.040045	Actin filament-coating protein tropomyosin [Handroanthus impetiginosus]
Daucus_carota_newGene_5220	-1.26394	PREDICTED: GDSL esterase/lipase At1g29670-like isoform X1 [Daucus carota subsp. sativus]
Daucus_carota_newGene_5221	3.616284	PREDICTED: GDSL esterase/lipase At1g29670-like [Daucus carota subsp. sativus]
Daucus_carota_newGene_5264	2.041882	--
Daucus_carota_newGene_5277	-Inf	--
Daucus_carota_newGene_5419	-3.28472	PREDICTED: subtilisin-like protease SBT1.1 [Vitis vinifera]
Daucus_carota_newGene_5772	2.548302	hypothetical protein DCAR_000801 [Daucus carota subsp. sativus]
Daucus_carota_newGene_5806	-4.35958	PREDICTED: uncharacterized protein LOC108197196 [Daucus carota subsp. sativus]
Daucus_carota_newGene_581	-2.87385	LRR receptor-like serine/threonine-protein kinase EFR [Capsicum chinense]
Daucus_carota_newGene_600	Inf	PREDICTED: uncharacterized protein LOC108216967 [Daucus carota subsp. sativus]
Daucus_carota_newGene_6007	-1.21197	PREDICTED: glu S.griseus protease inhibitor-like [Daucus carota subsp. sativus]

Daucus_carota_newGene_603	Inf	PREDICTED: vesicle-associated protein 1-1-like [Daucus carota subsp. sativus]
Daucus_carota_newGene_6033	2.142085	PREDICTED: G-type lectin S-receptor-like serine/threonine-protein kinase At4g27290 [Daucus carota subsp. sativus]
Daucus_carota_newGene_6252	-3.86655	PREDICTED: protein PHLOEM PROTEIN 2-LIKE A1-like [Daucus carota subsp. sativus]
Daucus_carota_newGene_6293	2.515199	PREDICTED: uncharacterized protein LOC108194185 [Daucus carota subsp. sativus]
Daucus_carota_newGene_6430	1.688109	PREDICTED: heat stress transcription factor A-4c-like [Daucus carota subsp. sativus]
Daucus_carota_newGene_6556	2.099031	PREDICTED: 13 kDa ribonucleoprotein-associated protein-like isoform X1 [Daucus carota subsp. sativus]
Daucus_carota_newGene_6646	-3.71737	PREDICTED: uncharacterized protein LOC108207462 [Daucus carota subsp. sativus]
Daucus_carota_newGene_6694	-2.14373	PREDICTED: beta-glucosidase BoGH3B-like [Daucus carota subsp. sativus]
Daucus_carota_newGene_6830	5.177468	PREDICTED: uncharacterized protein LOC108195084 [Daucus carota subsp. sativus]
Daucus_carota_newGene_7009	-1.23971	PREDICTED: glu S.griseus protease inhibitor-like [Daucus carota subsp. sativus]
Daucus_carota_newGene_7010	-1.82556	PREDICTED: proteinase inhibitor-like [Daucus carota subsp. sativus]
Daucus_carota_newGene_7121	1.516077	PREDICTED: anthocyanidin 3-O-glucosyltransferase 2-like [Daucus carota subsp. sativus]
Daucus_carota_newGene_7146	-6.06223	PREDICTED: abrin-b-like [Daucus carota subsp. sativus]
Daucus_carota_newGene_7147	-6.03334	PREDICTED: abrin-b-like [Daucus carota subsp. sativus]
Daucus_carota_newGene_7239	3.690536	PREDICTED: MADS-box transcription factor 23-like [Daucus carota subsp. sativus]
Daucus_carota_newGene_7331	1.00256	PREDICTED: TMV resistance protein N-like [Daucus carota subsp. sativus]
Daucus_carota_newGene_7432	3.336837	hypothetical protein DCAR_028902 [Daucus carota subsp. sativus]
Daucus_carota_newGene_7527	1.594726	PREDICTED: titin-like [Daucus carota subsp. sativus]
Daucus_carota_newGene_787	1.546759	PREDICTED: uncharacterized protein LOC108217000 [Daucus carota subsp. sativus]
Daucus_carota_newGene_854	2.258092	PREDICTED: peroxidase N1 [Ricinus communis]
Daucus_carota_newGene_947	-1.6774	--
Daucus_carota_newGene_95	1.456453	PREDICTED: uncharacterized protein LOC108222348 [Daucus carota subsp. sativus]
gene10035	-6.13529	unnamed protein product [Coffea canephora]
gene10036	-3.18953	PgMADS protein3 [Panax ginseng]
gene10075	-1.15021	unnamed protein product [Coffea canephora]
gene10503	-Inf	--
gene10505	-1.43132	Nodulin MtN3 family protein [Theobroma cacao]
gene10567	-2.20779	1-aminocyclopropane-1-carboxylate oxidase, putative [Ricinus communis]
gene10784	-2.58742	hypothetical protein JCGZ_12400 [Jatropha curcas]
gene10876	3.057889	PREDICTED: acetylajmalan esterase-like [Cucumis sativus]
gene10883	-1.81647	PREDICTED: stress-induced protein KIN2-like [Nicotiana sylvestris]
gene10937	2.164478	PREDICTED: serine carboxypeptidase-like 25-like [Solanum tuberosum]
gene10998	-1.69714	PREDICTED: vacuolar amino acid transporter 1-like [Fragaria vesca subsp. vesca]
gene11023	1.401795	PREDICTED: pleiotropic drug resistance protein 3 [Nicotiana sylvestris]
gene11046	-2.58411	PREDICTED: transcription factor bHLH135-like [Citrus sinensis]
gene11174	6.813824	PREDICTED: protein PLASTID MOVEMENT IMPAIRED 2 isoform X2 [Nelumbo nucifera]

gene11293	1.591649	unnamed protein product [Coffea canephora]
gene11378	-2.35605	--
gene11380	-2.58569	unnamed protein product [Coffea canephora]
gene11426	2.003125	dehydration responsive element-binding protein A6 [Daucus carota]
gene11427	-1.03926	PREDICTED: lipoxygenase homology domain-containing protein 1-like [Sesamum indicum]
gene11576	-1.2914	unnamed protein product [Coffea canephora]
gene11578	-2.52263	PREDICTED: 1-aminocyclopropane-1-carboxylate oxidase homolog 1-like [Fragaria vesca subsp. vesca]
gene11715	2.126718	PREDICTED: uncharacterized acetyltransferase At3g50280-like [Nicotiana tomentosiformis]
gene1175	4.125492	PREDICTED: LOW QUALITY PROTEIN: presequence protease 2, chloroplastic/mitochondrial-like [Citrus sinensis]
gene11775	1.916094	PREDICTED: abscisic acid receptor PYL4-like [Nicotiana tomentosiformis]
gene1181	-1.57781	PREDICTED: abscisic acid 8'-hydroxylase 4-like [Nicotiana sylvestris]
gene11862	-3.88554	PREDICTED: uncharacterized protein LOC100257848 [Vitis vinifera]
gene11870	1.48208	PREDICTED: serine/threonine-protein kinase At5g01020-like [Nicotiana sylvestris]
gene11884	-2.67751	PREDICTED: probable protein phosphatase 2C 50 isoform X2 [Nicotiana sylvestris]
gene11952	-1.05676	PREDICTED: acid phosphatase 1 [Prunus mume]
gene11968	-1.71796	unnamed protein product [Vitis vinifera]
gene1202	-2.59355	PREDICTED: L-ascorbate oxidase-like [Sesamum indicum]
gene12025	-1.14595	orf [Pisum sativum]
gene12073	-2.32473	hypothetical protein VITISV_035938 [Vitis vinifera]
gene1226	5.332059	PREDICTED: UBA and UBX domain-containing protein At4g15410 isoform X1 [Vitis vinifera]
gene12639	-2.54091	PREDICTED: uncharacterized protein LOC105170116 [Sesamum indicum]
gene12731	-3.6853	PREDICTED: uncharacterized protein LOC104900579 [Beta vulgaris subsp. vulgaris]
gene12732	-3.29547	PREDICTED: uncharacterized protein LOC104900579 [Beta vulgaris subsp. vulgaris]
gene12734	-3.62964	PREDICTED: 23 kDa jasmonate-induced protein-like [Beta vulgaris subsp. vulgaris]
gene12735	-3.5584	PREDICTED: 23 kDa jasmonate-induced protein-like [Beta vulgaris subsp. vulgaris]
gene1293	2.383516	cytochrome b6 (chloroplast) [Campanula americana]
gene12970	2.96102	PREDICTED: cannabidiolic acid synthase-like 1 [Sesamum indicum]
gene1333	1.919034	PREDICTED: protein EXORDIUM-like 2 [Nicotiana tomentosiformis]
gene13390	-1.3657	PREDICTED: allene oxide cyclase 3, chloroplastic-like [Populus euphratica]
gene13407	1.675271	PREDICTED: abscisic acid receptor PYL4-like [Nicotiana tomentosiformis]
gene13447	-1.06807	PREDICTED: uncharacterized protein LOC104111803 [Nicotiana tomentosiformis]
gene13470	1.960961	hypothetical protein PRUPE_ppa000225mg [Prunus persica]
gene13479	-1.96857	PREDICTED: transcription factor BTF3 homolog 4-like [Eucalyptus grandis]
gene13542	1.182164	PREDICTED: sulfate transporter 4.1, chloroplastic-like [Glycine max]
gene13568	5.446628	pathogenesis related gene 3 [Bupleurum kaoi]
gene13574	-1.18646	PREDICTED: uncharacterized protein LOC105176574 [Sesamum indicum]

gene13579	-1.45417	PREDICTED: uncharacterized protein LOC103930028 [Pyrus x bretschneideri]
gene13608	1.307475	PREDICTED: MATE efflux family protein 6 isoform X1 [Vitis vinifera]
gene13609	-2.14422	PREDICTED: MATE efflux family protein 6 isoform X1 [Vitis vinifera]
gene13611	-2.15509	PREDICTED: MATE efflux family protein 6-like [Malus domestica]
gene13615	3.1765	hypothetical protein MIMGU_mgv1a008243mg [Erythranthe guttata]
gene13621	-6.63188	PREDICTED: kinesin-like protein NACK1 [Nicotiana tomentosiformis]
gene13763	1.768641	UDP-glycosyltransferase 73C3 [Morus notabilis]
gene13777	-2.03173	PREDICTED: ABC transporter B family member 1 [Sesamum indicum]
gene13825	-2.66971	hypothetical protein JCGZ_15650 [Jatropha curcas]
gene13862	1.476789	PREDICTED: uncharacterized protein LOC104592576 [Nelumbo nucifera]
gene13896	1.838201	PREDICTED: EIN3-binding F-box protein 1-like [Sesamum indicum]
gene13903	-3.2898	Major facilitator superfamily protein isoform 2 [Theobroma cacao]
gene13927	1.750725	inwardly rectifying potassium channel subunit [Daucus carota]
gene13928	-3.85841	PREDICTED: 26S protease regulatory subunit 8 homolog A [Nicotiana tomentosiformis]
gene13987	-2.2851	PREDICTED: adiponectin receptor protein 2-like [Citrus sinensis]
gene13991	-3.15354	PREDICTED: monoglyceride lipase-like [Malus domestica]
gene13999	1.717666	hypothetical protein JCGZ_12025 [Jatropha curcas]
gene14055	3.825009	SNKR2GH6 protein [Solanum schenckii]
gene14162	1.316491	transcription factor DcERF1 [Daucus carota]
gene14202	-1.32651	PREDICTED: heat stress transcription factor B-2a-like [Sesamum indicum]
gene14226	-2.33284	PREDICTED: NAC domain-containing protein 45 [Sesamum indicum]
gene14227	-1.03985	PREDICTED: NAC domain-containing protein 45 [Sesamum indicum]
gene14276	1.046155	putative beta-ring carotene hydroxylase [Daucus carota subsp. sativus]
gene14303	-1.16978	PREDICTED: transcription factor TCP20 [Vitis vinifera]
gene14315	-Inf	--
gene14332	1.92648	PREDICTED: cytochrome b-c1 complex subunit Rieske-4, mitochondrial-like [Pyrus x bretschneideri]
gene14364	-Inf	glucosyltransferase, partial [Gymnema sylvestre]
gene14529	-2.29368	PREDICTED: 3-phosphoinositide-dependent protein kinase 2 [Nicotiana sylvestris]
gene14631	1.997072	Receptor-like kinase 1 [Theobroma cacao]
gene14700	1.084778	PREDICTED: junctophilin-1-like [Solanum lycopersicum]
gene15000	-1.21132	Uncharacterized protein TCM_001721 [Theobroma cacao]
gene15015	-1.72669	putative 9-cis epoxycarotenoid dioxygenase [Daucus carota subsp. sativus]
gene1503	Inf	PREDICTED: uncharacterized protein LOC104415567 [Eucalyptus grandis]
gene15228	1.390522	Guanylate kinase [Gossypium arboreum]
gene15464	1.287612	Uncharacterized protein TCM_034800 [Theobroma cacao]
gene15754	1.533443	PREDICTED: ammonium transporter 3 member 3-like [Sesamum indicum]
gene15832	-1.16497	PREDICTED: probable indole-3-acetic acid-amido synthetase GH3.5 isoform X1 [Populus euphratica]
gene1618	1.232767	unnamed protein product [Coffea canephora]

gene16492	-1.34773	PREDICTED: cytochrome P450 71A1-like [Vitis vinifera]
gene16585	-2.36366	PREDICTED: protein NRT1/ PTR FAMILY 2.11-like [Nicotiana sylvestris]
gene16592	1.327978	unnamed protein product [Coffea canephora]
gene16600	-1.76126	PREDICTED: protein PHYTOCHROME KINASE SUBSTRATE 3-like [Nicotiana tomentosiformis]
gene16605	1.285929	PREDICTED: uncharacterized protein LOC100260374 [Vitis vinifera]
gene16640	-1.05295	Uncharacterized protein TCM_014112 [Theobroma cacao]
gene16653	-1.69062	MLO-like protein 4 [Morus notabilis]
gene16747	2.093683	RecName: Full=Glycine-rich protein DC9.1 [Daucus carota]
gene16748	3.681288	PREDICTED: putative receptor-like protein kinase At3g47110 [Elaeis guineensis]
gene16797	-1.22485	Expansin-A1 [Gossypium arboreum]
gene16948	-2.11722	unnamed protein product [Coffea canephora]
gene16970	4.147841	PREDICTED: calcium-dependent protein kinase 20-like [Cucumis sativus]
gene17306	1.452682	unnamed protein product [Vitis vinifera]
gene17920	2.128976	PREDICTED: EIN3-binding F-box protein 1-like [Sesamum indicum]
gene18052	1.189812	PREDICTED: UDP-glucuronate 4-epimerase 1-like [Glycine max]
gene18057	1.190414	PREDICTED: protein ECERIFERUM 3 [Vitis vinifera]
gene18083	1.645142	PREDICTED: probable xyloglucan endotransglucosylase/hydrolase protein 23 [Vitis vinifera]
gene18107	-1.5601	unknown [Populus trichocarpa]
gene18112	-1.74325	unnamed protein product [Coffea canephora]
gene18136	-2.75408	PREDICTED: transcription factor bHLH35-like isoform X1 [Nicotiana sylvestris]
gene18137	-4.27391	PREDICTED: transcription factor bHLH35 [Vitis vinifera]
gene18209	1.502439	hypothetical protein JCGZ_26281 [Jatropha curcas]
gene18221	2.079193	PREDICTED: 21 kDa protein [Vitis vinifera]
gene18244	1.590379	PREDICTED: uncharacterized protein LOC100265251 [Vitis vinifera]
gene18254	-1.24236	hypothetical protein PRUPE_ppa003851mg [Prunus persica]
gene1845	-1.14262	PREDICTED: probable pectate lyase 15-like [Fragaria vesca subsp. vesca]
gene18517	-2.24445	PREDICTED: uncharacterized protein At3g15000, mitochondrial-like [Tarenaya hassleriana]
gene18612	1.071199	unnamed protein product [Coffea canephora]
gene18884	1.54064	PREDICTED: uncharacterized acetyltransferase At3g50280-like [Citrus sinensis]
gene18950	-1.45466	hypothetical protein PRUPE_ppa013763mg [Prunus persica]
gene19108	-1.08883	PREDICTED: uncharacterized protein LOC101250947 [Solanum lycopersicum]
gene1918	-1.21034	PREDICTED: cathepsin B [Brassica rapa]
gene19209	-2.39833	PREDICTED: glucomannan 4-beta-mannosyltransferase 9 [Vitis vinifera]
gene19210	-5.58957	hypothetical protein JCGZ_01530 [Jatropha curcas]
gene19211	-2.08686	hypothetical protein JCGZ_01530 [Jatropha curcas]
gene19354	4.04189	PREDICTED: uncharacterized protein LOC104903079 [Beta vulgaris subsp. vulgaris]
gene19358	-1.096	PREDICTED: expansin-B3-like [Eucalyptus grandis]
gene19381	-1.5274	hypothetical protein JCGZ_14941 [Jatropha curcas]

gene19598	1.239941	unnamed protein product [Coffea canephora]
gene19685	2.965582	unnamed protein product [Coffea canephora]
gene19808	3.791024	hypothetical protein Csa_5G611020 [Cucumis sativus]
gene19908	1.243086	PREDICTED: uncharacterized protein LOC104242669 isoform X4 [Nicotiana sylvestris]
gene20040	3.204638	PREDICTED: abietadienol/abietadienal oxidase [Nicotiana sylvestris]
gene20058	2.736899	PREDICTED: geraniol 8-hydroxylase-like [Solanum tuberosum]
gene20120	1.488824	PREDICTED: cytochrome P450 78A5-like [Nicotiana sylvestris]
gene20169	4.464473	PREDICTED: GDSL esterase/lipase At4g16230-like [Cucumis melo]
gene2029	-1.00201	hypothetical protein CICLE_v10005298mg [Citrus clementina]
gene20330	-1.57664	cyclophilin [Daucus carota]
gene20432	-2.71497	PREDICTED: U-box domain-containing protein 7-like [Prunus mume]
gene2065	3.202517	transcription factor DcERF2 [Daucus carota]
gene2069	-1.0231	RecName: Full=Major allergen Api g 1, isoallergen 2; AltName: Full=Allergen Api g 1.0201; AltName: Allergen=Api g 1 [Apium graveolens]
gene20862	2.254741	PREDICTED: phylloplanin [Vitis vinifera]
gene20864	3.344368	PREDICTED: LOW QUALITY PROTEIN: phylloplanin-like [Pyrus x bretschneideri]
gene2094	Inf	PREDICTED: peroxidase 29-like [Malus domestica]
gene21241	-1.27681	PREDICTED: CDPK-related kinase 5 [Nelumbo nucifera]
gene21308	1.208952	PREDICTED: probable LRR receptor-like serine/threonine-protein kinase At4g36180 [Solanum lycopersicum]
gene21349	-1.18252	Vaculolar sorting receptor 3 isoform 1 [Theobroma cacao]
gene21375	3.46569	hypothetical protein CICLE_v10029873mg [Citrus clementina]
gene21419	-1.4571	PREDICTED: root phototropism protein 3-like [Nicotiana tomentosiformis]
gene21425	-1.40193	unnamed protein product [Coffea canephora]
gene21431	1.113608	hypothetical protein L484_017174 [Morus notabilis]
gene21470	-1.84265	unnamed protein product [Coffea canephora]
gene21535	-1.18886	Chloride channel CLC-b -like protein [Gossypium arboreum]
gene21536	1.028388	PREDICTED: uncharacterized protein LOC100816889 [Glycine max]
gene21587	-2.18428	PREDICTED: probable N-acetyl-gamma-glutamyl-phosphate reductase, chloroplastic isoform X3 [Elaeis guineensis]
gene21605	1.204458	PREDICTED: high affinity nitrate transporter 2.5 [Vitis vinifera]
gene21634	1.212514	PREDICTED: L-aspartate oxidase, chloroplastic [Sesamum indicum]
gene21726	-2.59108	hypothetical protein JCGZ_11376 [Jatropha curcas]
gene21728	-3.56947	hypothetical protein JCGZ_11393 [Jatropha curcas]
gene21880	-1.51137	unnamed protein product [Coffea canephora]
gene2204	3.078204	PREDICTED: deacetylvindoline O-acetyltransferase-like [Solanum tuberosum]
gene2205	1.853078	PREDICTED: BAHD acyltransferase At5g47980-like [Prunus mume]
gene22124	2.052769	PREDICTED: malonyl-coenzyme:anthocyanin 5-O-glucoside-6'''-O-malonyltransferase {ECO:0000303|PubMed:11598135}-like [Nicotiana tomentosiformis]

gene22127	-2.94834	quercetin 3-O-glucoside-6''-O-malonyltransferase [Glandularia x hybrida]
gene2224	2.419714	PREDICTED: putative protease Do-like 14 isoform X1 [Nicotiana tomentosiformis]
gene22257	4.364113	PREDICTED: receptor-like protein 12 [Vitis vinifera]
gene22272	1.923422	unnamed protein product [Coffea canephora]
gene22370	2.123422	hypothetical protein MVEG_09432 [Mortierella verticillata NRRL 6337]
gene2242	4.330678	PREDICTED: type I inositol 1,4,5-trisphosphate 5-phosphatase CVP2-like isoform X1 [Nicotiana tomentosiformis]
gene22425	1.724911	unnamed protein product [Coffea canephora]
gene22599	-1.23977	PREDICTED: enolase [Sesamum indicum]
gene22686	2.247851	Plant U-box 23 [Theobroma cacao]
gene22736	-5.18471	hypothetical protein POPTR_0013s06280g [Populus trichocarpa]
gene22771	1.413261	bZIP transcription factor bZIP7 [Camellia sinensis]
gene22791	5.733186	hypothetical protein PRUPE_ppa005914mg [Prunus persica]
gene2281	1.217463	ATPase E1-E2 type family protein / haloacid dehalogenase-like hydrolase family protein [Theobroma cacao]
gene22816	1.199206	PREDICTED: vacuolar amino acid transporter 1-like isoform X1 [Citrus sinensis]
gene22817	-5.3774	PREDICTED: vacuolar amino acid transporter 1-like isoform X1 [Eucalyptus grandis]
gene22824	2.876667	hypothetical protein POPTR_0016s03640g [Populus trichocarpa]
gene22909	1.22666	JMS10C05.2 [Jatropha curcas]
gene22963	-2.76106	unnamed protein product [Coffea canephora]
gene23004	1.287087	PREDICTED: NAC domain-containing protein 45 [Sesamum indicum]
gene23036	-2.21485	PREDICTED: probable 6-phosphogluconolactonase 4, chloroplastic isoform X2 [Nelumbo nucifera]
gene23050	1.22323	hypothetical protein JCGZ_08143 [Jatropha curcas]
gene23079	3.510601	pathogenesis related gene 5 [Bupleurum kaoi]
gene23147	-2.83662	PREDICTED: uncharacterized protein LOC101293047 [Fragaria vesca subsp. vesca]
gene23218	1.111446	PREDICTED: U-box domain-containing protein 35-like isoform X1 [Vitis vinifera]
gene23280	1.174272	unnamed protein product [Vitis vinifera]
gene23438	2.36555	PREDICTED: probable WRKY transcription factor 51 [Nelumbo nucifera]
gene2347	2.473085	PREDICTED: probable galacturonosyltransferase-like 4 [Sesamum indicum]
gene23528	-1.38929	hypothetical protein ARALYDRAFT_345475 [Arabidopsis lyrata subsp. lyrata]
gene23615	-1.95785	Arginine methyltransferase 4A isoform 1 [Theobroma cacao]
gene23623	1.040824	unnamed protein product [Vitis vinifera]
gene23690	-3.27147	PREDICTED: cell wall / vacuolar inhibitor of fructosidase 1-like [Prunus mume]
gene23754	-1.38906	PREDICTED: uncharacterized protein LOC102600609 [Solanum tuberosum]
gene23768	2.680427	photosystem II cp47 protein, partial (chloroplast) [Abrophyllum ornans]
gene2380	1.150357	PREDICTED: O-glucosyltransferase rumi homolog isoform X2 [Vitis vinifera]
gene23926	1.342106	PREDICTED: pectinesterase-like [Nelumbo nucifera]
gene2398	-1.78206	hypothetical protein CICLE_v10023157mg [Citrus clementina]
gene2419	-1.24942	(3S)-linalool/(E)-nerolidol synthase [Vitis vinifera]

gene2438	1.430301	PREDICTED: glyoxysomal fatty acid beta-oxidation multifunctional protein MFP-a [Nicotiana sylvestris]
gene2441	5.347514	Cytochrome P450 [Theobroma cacao]
gene2452	2.218112	ethylene receptor ERS1b [Actinidia deliciosa]
gene24605	-3.88061	RecName: Full=Proline-rich 33 kDa extensin-related protein; Flags: Precursor, partial [Daucus carota]
gene24639	-1.99642	PREDICTED: LOW QUALITY PROTEIN: phosphate transporter PHO1 [Sesamum indicum]
gene24704	-1.21975	unknown [Lotus japonicus]
gene24757	-1.64207	PREDICTED: linoleate 13S-lipoxygenase 2-1, chloroplastic-like [Citrus sinensis]
gene2490	1.84162	PREDICTED: universal stress protein A-like protein [Sesamum indicum]
gene25487	-1.24566	PREDICTED: ethylene-responsive transcription factor 13-like [Solanum lycopersicum]
gene2554	3.059912	cytosolic glucose-6-phosphate dehydrogenase 2 [Petroselinum crispum]
gene25576	1.25098	PREDICTED: glyoxylate/hydroxypyruvate reductase HPR3 isoform X2 [Vitis vinifera]
gene25581	-2.45136	PREDICTED: putative disease resistance protein RGA1 [Prunus mume]
gene25689	1.111149	UGT6 [Panax ginseng]
gene2572	1.667625	PREDICTED: 3-ketoacyl-CoA synthase 1 [Vitis vinifera]
gene25756	-1.02722	PREDICTED: hydrophobic protein RCI2B-like [Brassica rapa]
gene25757	1.820559	Putative peptide/nitrate transporter [Glycine soja]
gene25899	1.002885	hypothetical protein POPTR_0017s13240g [Populus trichocarpa]
gene26250	-1.19998	PREDICTED: LOB domain-containing protein 37 [Solanum lycopersicum]
gene26311	1.211861	phosphoenolpyruvate carboxykinase [Flaveria pringlei]
gene26353	3.220882	PREDICTED: uncharacterized protein LOC105174719 [Sesamum indicum]
gene26436	-1.42863	Salicylate O-methyltransferase [Theobroma cacao]
gene26605	-1.22188	PREDICTED: probable polygalacturonase non-catalytic subunit JP650 [Sesamum indicum]
gene26682	-1.42428	hypothetical protein F383_28754 [Gossypium arboreum]
gene26920	2.140498	PREDICTED: cation/calcium exchanger 4-like [Nicotiana tomentosiformis]
gene27008	6.695929	PREDICTED: lipoyl synthase 1, mitochondrial-like [Solanum lycopersicum]
gene27025	1.560236	unnamed protein product [Coffea canephora]
gene27029	1.177056	unnamed protein product [Coffea canephora]
gene27160	1.469703	PREDICTED: leucine-rich repeat extensin-like protein 4 [Brassica rapa]
gene27205	Inf	PsbA [Dianthus sylvestris]
gene27317	-2.4034	PREDICTED: protein SRG1-like [Sesamum indicum]
gene27372	-1.20719	PREDICTED: phytosulfokines-like [Nicotiana tomentosiformis]
gene27397	-1.69273	unnamed protein product [Vitis vinifera]
gene27443	1.057443	PREDICTED: uncharacterized protein LOC104228445 [Nicotiana sylvestris]
gene2769	-1.07778	PREDICTED: probable serine/threonine-protein kinase RLCKVII [Nicotiana sylvestris]
gene27830	-1.12887	PREDICTED: bifunctional epoxide hydrolase 2-like [Sesamum indicum]
gene27896	-1.80207	uridine diphosphate glycosyltransferase [Bupleurum chinense]

gene28172	-2.41465	--
gene28173	-4.47053	--
gene28174	-4.9863	--
gene28181	1.660342	PREDICTED: aspartic proteinase nepenthesin-1 [Nicotiana sylvestris]
gene28379	-1.42226	PREDICTED: protein phosphatase 2C 37 [Solanum lycopersicum]
gene28553	-1.48046	PREDICTED: expansin-A1 [Populus euphratica]
gene28554	-1.87528	PREDICTED: expansin-A1 [Populus euphratica]
gene28723	-1.15592	PREDICTED: uncharacterized protein LOC100251040 [Vitis vinifera]
gene28795	1.071133	PREDICTED: protein TIME FOR COFFEE isoform X3 [Nicotiana sylvestris]
gene2881	1.603983	PREDICTED: protein NRT1/ PTR FAMILY 4.3-like [Sesamum indicum]
gene28811	1.000847	PREDICTED: protein kinase APK1B, chloroplastic [Vitis vinifera]
gene28832	1.271891	PREDICTED: keratin, type I cytoskeletal 9-like [Nicotiana tomentosiformis]
gene28891	2.200839	hypothetical protein CICLE_v10005161mg [Citrus clementina]
gene28898	2.75207	--
gene28922	2.484084	Pollen Ole e 1 allergen and extensin family protein, putative [Theobroma cacao]
gene28925	6.855143	PREDICTED: phylloplanin-like [Populus euphratica]
gene28965	-2.0098	PREDICTED: GATA transcription factor 15-like isoform X2 [Nicotiana sylvestris]
gene28966	-1.06698	PREDICTED: uncharacterized protein LOC104108341 [Nicotiana tomentosiformis]
gene29124	-2.08351	unnamed protein product [Vitis vinifera]
gene29210	1.805609	sulfate transporter, putative [Ricinus communis]
gene29213	2.9097	BnaC06g32110D [Brassica napus]
gene29214	2.502166	PREDICTED: chitin-binding lectin 1 [Vitis vinifera]
gene29219	1.562045	PREDICTED: U-box domain-containing protein 11-like [Sesamum indicum]
gene29248	-Inf	PREDICTED: flavonol 3-sulfotransferase-like [Populus euphratica]
gene29252	-1.13886	PREDICTED: chaperone protein dnaJ 15-like isoform X1 [Citrus sinensis]
gene29265	-1.22795	alpha-glucosidase, putative [Ricinus communis]
gene29266	-1.68253	PREDICTED: uncharacterized protein LOC104104866 [Nicotiana tomentosiformis]
gene29279	1.781431	tempranillo [Olea europaea]
gene29302	-4.73366	PREDICTED: L-ascorbate oxidase-like [Sesamum indicum]
gene29347	2.008328	PREDICTED: LOW QUALITY PROTEIN: disease resistance protein RPP13-like [Nicotiana tomentosiformis]
gene29402	-1.27579	unnamed protein product [Coffea canephora]
gene29455	1.362671	xyloglucan endotransglycosylase hydrolase [Apium graveolens]
gene29620	1.186306	PREDICTED: probable LRR receptor-like serine/threonine-protein kinase At2g16250 [Sesamum indicum]
gene29658	Inf	unnamed protein product [Coffea canephora]
gene29696	2.197297	PREDICTED: glycoprotein 3-alpha-L-fucosyltransferase A-like [Nicotiana sylvestris]
gene29811	-2.09586	PREDICTED: GDSL esterase/lipase At5g03980-like isoform X2 [Solanum lycopersicum]
gene29813	2.456465	hypothetical protein VITISV_024777 [Vitis vinifera]

gene29823	-4.40276	PREDICTED: cyanidin-3-O-glucoside 2-O-glucuronosyltransferase-like [Prunus mume]
gene29846	1.419519	unnamed protein product [Coffea canephora]
gene29875	-2.01497	hypothetical protein AALP_AA1G269100 [Arabis alpina]
gene30019	-2.71234	Basic helix-loop-helix DNA-binding superfamily protein isoform 2 [Theobroma cacao]
gene30067	-3.79885	PREDICTED: lysine-specific demethylase JMJ25-like [Sesamum indicum]
gene30114	1.257198	PREDICTED: keratin, type I cytoskeletal 9-like [Eucalyptus grandis]
gene30136	2.40091	PREDICTED: uncharacterized protein LOC104897274 isoform X2 [Beta vulgaris subsp. vulgaris]
gene30192	-1.14752	PREDICTED: probable receptor-like protein kinase At5g47070-like isoform X3 [Solanum tuberosum]
gene30227	-2.32534	S-adenosyl-L-methionine-dependent methyltransferases superfamily protein, putative [Theobroma cacao]
gene30327	-1.79964	PREDICTED: pathogenesis-related protein 5-like [Vitis vinifera]
gene30364	1.063121	hypothetical protein L484_012503 [Morus notabilis]
gene3075	-1.48353	PREDICTED: uncharacterized protein LOC100251922 [Vitis vinifera]
gene30818	1.956034	PREDICTED: AP2/ERF and B3 domain-containing transcription factor RAV1-like [Vitis vinifera]
gene31114	-3.21092	PREDICTED: cytosolic sulfotransferase 5-like [Populus euphratica]
gene31122	2.417814	hypothetical protein MIMGU_mgv1a025785mg [Erythranthe guttata]
gene31262	2.662039	PREDICTED: phylloplanin [Vitis vinifera]
gene31264	3.247204	PREDICTED: phylloplanin [Tarenaya hassleriana]
gene3142	3.760155	PREDICTED: protein NRT1/ PTR FAMILY 5.10-like [Nicotiana sylvestris]
gene3143	1.100752	PREDICTED: mitogen-activated protein kinase kinase 9-like [Nelumbo nucifera]
gene31460	-1.63781	PREDICTED: uncharacterized protein LOC100251040 [Vitis vinifera]
gene31492	1.364514	ethylene receptor ETR2 [Actinidia deliciosa]
gene31576	1.280136	beta-galactosidase [Camellia sinensis]
gene31769	-2.26052	PREDICTED: expansin-A1 [Populus euphratica]
gene31836	-1.34236	PREDICTED: tetratricopeptide repeat protein 38-like [Solanum tuberosum]
gene31852	2.362272	PREDICTED: E3 ubiquitin-protein ligase TRAF7-like [Nicotiana sylvestris]
gene31997	1.416274	PREDICTED: vacuolar protein sorting-associated protein 41 homolog [Malus domestica]
gene32020	-2.24968	PREDICTED: protein SRG1-like [Sesamum indicum]
gene32129	Inf	PREDICTED: flavonol synthase/flavanone 3-hydroxylase-like [Nicotiana tomentosiformis]
gene32272	3.041885	hypothetical protein MIMGU_mgv1a023987mg [Erythranthe guttata]
gene32279	-1.20688	PREDICTED: U-box domain-containing protein 72 [Solanum lycopersicum]
gene32457	-1.36386	profilin 3 [Petroselinum crispum]
gene3265	1.727968	PREDICTED: DNA replication licensing factor MCM4 [Nicotiana sylvestris]
gene32663	-3.58183	hypothetical protein JCGZ_11393 [Jatropha curcas]
gene32741	-2.0655	unnamed protein product [Vitis vinifera]
gene3282	1.72427	DnaJ homolog subfamily C member 2 [Morus notabilis]

gene32998	-1.51267	plasma membrane intrinsic protein [Olea europaea]
gene33314	2.135211	photosystem II protein D1 [Arabidopsis thaliana]
gene33327	2.088899	ATP synthase CF0 subunit IV [Daucus carota]
gene33330	3.236487	RNA polymerase beta [Daucus carota]
gene33331	2.476871	RNA polymerase beta subunit [Daucus carota]
gene33339	2.463812	photosystem II protein D2 [Daucus carota]
gene33340	2.396876	photosystem II CP43 chlorophyll apoprotein [Daucus carota]
gene33342	2.637627	photosystem II protein Z [Daucus carota]
gene33346	2.635838	photosystem I P700 apoprotein A2 [Anthriscus cerefolium]
gene33347	2.779902	photosystem I P700 apoprotein A1 [Daucus carota]
gene33361	2.198839	ribulose 1,5-bisphosphate carboxylase/oxygenase large subunit [Daucus carota]
gene33364	2.361589	photosystem I assembly protein ycf4 [Daucus carota]
gene33365	2.069457	envelope membrane protein [Daucus carota]
gene33366	1.442589	cytochrome f [Daucus carota]
gene33382	1.850929	photosystem II CP47 chlorophyll apoprotein [Daucus carota]
gene33385	2.294393	photosystem II phosphoprotein [Daucus carota]
gene33386	1.864736	cytochrome b6 [Panax ginseng]
gene33395	2.371184	ribosomal protein S3 [Daucus carota]
gene33415	2.055876	NADH-plastoquinone oxidoreductase subunit 5 [Daucus carota]
gene33418	2.289892	cytochrome c heme attachment protein [Daucus carota]
gene33419	2.047878	NADH-plastoquinone oxidoreductase subunit 4 [Daucus carota]
gene33424	2.469029	NADH-plastoquinone oxidoreductase subunit 1 [Daucus carota]
gene33425	2.360123	NADH-plastoquinone oxidoreductase subunit 7 [Daucus carota]
gene3535	-2.47774	hypothetical protein VITISV_005574 [Vitis vinifera]
gene3583	1.629821	PREDICTED: subtilisin-like protease SBT5.3 [Nicotiana tomentosiformis]
gene3817	-1.1484	PREDICTED: probable UDP-3-O-acylglucosamine N-acyltransferase 2, mitochondrial isoform X2 [Beta vulgaris subsp. vulgaris]
gene3940	2.369206	hypothetical protein POPTR_0006s01360g [Populus trichocarpa]
gene397	-1.08377	BnaA01g10370D [Brassica napus]
gene400	-Inf	Kinesin-like protein 1 isoform 2 [Theobroma cacao]
gene4050	1.863918	PREDICTED: TMV resistance protein N-like [Nicotiana tomentosiformis]
gene4137	1.047765	PREDICTED: myb-related protein 3R-1 [Nicotiana sylvestris]
gene4165	1.11012	PREDICTED: ABC transporter G family member 14 [Vitis vinifera]
gene4178	2.204143	cytochrome P450 CYP707A67 [Bupleurum chinense]
gene4218	-2.18066	unnamed protein product [Coffea canephora]
gene4220	6.317581	unnamed protein product [Coffea canephora]
gene4221	6.196684	unnamed protein product [Coffea canephora]
gene4258	2.957109	hypothetical protein PRUPE_ppa022610mg [Prunus persica]
gene4348	-1.11579	PREDICTED: uncharacterized protein LOC104903079 [Beta vulgaris subsp. vulgaris]
gene4398	-3.89225	PREDICTED: peptidyl-prolyl cis-trans isomerase FKBP62-like isoform X1 [Eucalyptus grandis]

gene4401	3.0176	PREDICTED: quinone-oxidoreductase homolog, chloroplastic-like [Malus domestica]
gene4516	1.11703	PREDICTED: uncharacterized membrane protein At1g16860-like [Solanum tuberosum]
gene4613	-3.09698	unnamed protein product [Daucus carota]
gene5011	-1.01334	metallothionein-like protein [Panax ginseng]
gene5051	1.203694	PREDICTED: GDSL esterase/lipase At4g01130-like [Sesamum indicum]
gene5377	-2.06659	PREDICTED: protein LIGHT-DEPENDENT SHORT HYPOCOTYLS 4-like [Sesamum indicum]
gene5592	-1.32995	PREDICTED: uncharacterized protein LOC104120793 [Nicotiana tomentosiformis]
gene5675	-1.46685	hypothetical protein CICLE_v10029517mg [Citrus clementina]
gene5761	-1.09947	RecName: Full=Elongation factor 1-alpha; Short=EF-1-alpha [Daucus carota]
gene5815	2.988222	PREDICTED: high affinity nitrate transporter 2.3-like [Sesamum indicum]
gene582	1.01681	PREDICTED: serpin-ZX-like isoform X3 [Solanum lycopersicum]
gene5990	1.89995	PREDICTED: protein EXORDIUM-like 5 [Nelumbo nucifera]
gene5999	1.297606	PREDICTED: protein NLP2-like [Nicotiana tomentosiformis]
gene6490	1.021367	PREDICTED: SPX domain-containing membrane protein At4g22990-like isoform X2 [Nicotiana sylvestris]
gene6628	-1.29298	unnamed protein product [Coffea canephora]
gene6683	1.165946	delta-8 sphingolipid desaturase [Crepis alpina]
gene6974	1.691764	PREDICTED: AP2/ERF and B3 domain-containing transcription factor RAV1-like [Vitis vinifera]
gene6992	-1.04059	PREDICTED: uncharacterized protein LOC101254384 [Solanum lycopersicum]
gene7238	-1.01755	pectate lyase [Gossypium laxum]
gene7251	-2.81145	PREDICTED: uncharacterized protein LOC102590433 [Solanum tuberosum]
gene7273	1.368427	PREDICTED: exocyst complex component EXO70B1 [Vitis vinifera]
gene7284	3.017051	unnamed protein product [Coffea canephora]
gene7323	1.412368	PDH2 [Helianthus tuberosus]
gene7354	-2.51651	Alcohol dehydrogenase transcription factor Myb/SANT-like family protein [Theobroma cacao]
gene7402	2.908546	PREDICTED: transcription factor bHLH87 [Populus euphratica]
gene7457	-1.93095	JAZ2 [Hevea brasiliensis]
gene7685	-1.15279	--
gene7754	1.178444	PREDICTED: probable LRR receptor-like serine/threonine-protein kinase At2g24230 [Vitis vinifera]
gene7806	1.415522	PREDICTED: glycerophosphodiester phosphodiesterase protein kinase domain-containing GDPDL2-like [Vitis vinifera]
gene7807	1.238991	unnamed protein product [Coffea canephora]
gene7828	-Inf	--
gene7935	2.083302	hypothetical protein PRUPE_ppa002615mg [Prunus persica]
gene801	-1.04017	PREDICTED: uncharacterized protein LOC100853163 isoform X1 [Vitis vinifera]
gene811	-2.46688	t-anol/isoeugenol synthase [Pimpinella anisum]
gene8348	1.037101	Aluminum-activated malate transporter 9 [Theobroma cacao]

gene858	1.01045	PREDICTED: DEAD-box ATP-dependent RNA helicase 39 [Vitis vinifera]
gene8628	1.323807	PREDICTED: patatin-like protein 2 isoform X2 [Solanum lycopersicum]
gene8711	2.426634	unnamed protein product [Coffea canephora]
gene8960	-3.86934	unnamed protein product [Coffea canephora]
gene9029	1.73542	unnamed protein product [Coffea canephora]
gene9074	-1.6601	type 2 metallothionein [Arachis hypogaea]
gene946	-3.1833	PREDICTED: linoleate 13S-lipoxygenase 2-1, chloroplastic-like [Citrus sinensis]
gene9675	-2.37733	PREDICTED: uncharacterized protein LOC105178714 [Sesamum indicum]
gene970	3.284243	PREDICTED: G-type lectin S-receptor-like serine/threonine-protein kinase RLK1 isoform X1 [Vitis vinifera]
gene9708	1.588498	PREDICTED: uncharacterized protein LOC105158850 [Sesamum indicum]
gene9714	-1.81241	bZIP transcription factor protein [Capsicum annuum]
gene9762	1.498334	unnamed protein product [Vitis vinifera]
gene9804	-Inf	unnamed protein product [Vitis vinifera]
gene9944	4.99045	hypothetical protein M569_12652, partial [Genlisea aurea]
gene9973	-1.32231	hydrolase, putative [Ricinus communis]
