# Supplementary material for: The genes crucial to carotenoid metabolism under elevated CO2 levels in carrot (Daucus carota L.)
Source: Sci Rep. 2021 Jun 8;11:12073. doi: 10.1038/s41598-021-91522-7 (PMC8187729; doi:10.1038/s41598-021-91522-7)
Supplement: Supplementary file 2 — Supplementary Information 2. [file 41598_2021_91522_MOESM2_ESM.pdf]

# The Genes Crucial to Carotenoid Metabolism in Carrot (*Daucus carota* L.) under Elevated $CO_2$ Levels

Hongxia Song[†], Qiang Lu[†], Leiping Hou and Meilan Li*

Collaborative Innovation Center for Improving Quality and Increasing Profits of Protected Vegetables in Shanxi, College of Horticulture, Shanxi Agricultural University, Taigu, Shanxi, China

[†]These authors have contributed equally to this work

[*] Corresponding author:

Prof. Meilan Li.

E-mail: 15935485975@163.com

Institutional mail: College of Horticulture, Shanxi Agricultural University, Taigu District, Shanxi Province, P. R. China. Zip code: 030801

**Supplementary Table S2 Primers used for RT-qPCR analysis.**

| Gene ID | Forward primer (5'–3') | Reverse primer (5'–3') |
|---|---|---|
| *gene14276* | TCTAGCTCACCTCTCGGTAATC | CAGTCATCTGTTTGGGAACCT |
| *gene15015* | GCAGGTACACTGAAACAGAGAG | CAAGTCGAGCTATCCCTGAATG |
| *gene4178* | GTGTGCACTGAGCTGTATGA | AGAGTGTTGGTTTGGAGAGTG |
| *gene1181* | GGTCCTTCTGCACTGTTCTT | ATGGCTGTGGCCTCAATATC |
| *gene24757* | AGATCGGAGGTGTGAAAGTAGA | TGACAAGGATGCTGCCATTAG |
| *gene946* | CGTTCGCGGAGAAAGTAGTAA | CACCGTCGTCGTGATCTTAAT |
| *gene33346* | ATGCCGAATCTCGTCTCAATC | CAGGAATAGCCACGTGTACTAAA |
| *gene33340* | TGGTCTGGAGAGGCTTACTT | CGCTAGGATAGGCGGTATTATTG |
| *gene2438* | GCCACGGAATCTCCATGTTA | GACCTCCAGAACGGTCTTATTC |
| *gene13390* | CCAAGAGTTGTCGGTGTATGAA | GGAACGAGATCACCCAAAGAG |
| *Actin* | GCGGGAAATTGTTCGTGATATG | CCCATCAGGCAGTTCATAGTT |
